# Metabolic engineering of *Synechocystis* sp. PCC 6803 for improved bisabolene production

**DOI:** 10.1016/j.mec.2020.e00159

**Published:** 2020-12-25

**Authors:** João S. Rodrigues, Pia Lindberg

**Affiliations:** Department of Chemistry – Ångström, Uppsala University, Uppsala, Sweden

**Keywords:** Metabolic engineering, Bisabolene, Synechocystis, MEP pathway, High cell density

## Abstract

Terpenoids are a wide class of organic compounds with industrial relevance. The natural ability of cyanobacteria to produce terpenoids via the methylerythritol 4-phosphate (MEP) pathway makes these organisms appealing candidates for the generation of light-driven cell factories for green chemistry. Here we address the improvement of the production of (*E*)-α-bisabolene, a valuable biofuel feedstock, in *Synechocystis* sp. PCC 6803 via sequential heterologous expression of bottleneck enzymes of the native pathway. Expression of the bisabolene synthase is sufficient to complete the biosynthetic pathway of bisabolene. Expression of a farnesyl-pyrophosphate synthase from *Escherichia coli* did not influence production of bisabolene, while enhancement of the MEP pathway via additional overexpression of 1-deoxy-*D*-xylulose-5-phosphate synthase (DXS) and IPP/DMAPP isomerase (IDI) significantly increased production per cell. However, in the absence of a carbon sink, the overexpression of DXS and IDI leads to significant growth impairment. The final engineered strain reached a volumetric titre of 9 ​mg ​L^−1^ culture of bisabolene after growing for 12 days. When the cultures were grown in a high cell density (HCD) system, we observed an increase in the volumetric titres by one order of magnitude for all producing-strains. The strain with improved MEP pathway presented an increase twice as much as the remaining engineered strains, yielding more than 180 ​mg ​L^−1^ culture after 10 days of cultivation. Furthermore, the overexpression of these two MEP enzymes prevented the previously reported decrease in the bisabolene specific titres when grown in HCD conditions, where primary metabolism is usually favoured. We conclude that fine-tuning of the cyanobacterial terpenoid pathway is crucial for the generation of microbial platforms for terpenoid production on industrial-scale.

## Highlights

• Overexpressing two bottleneck enzymes from MEP pathway doubles bisabolene titres.
• Enhancing MEP pathway in the absence of a proper carbon sink compromised growth.
• Growth of bisabolene-producing strains in HDC system increased titres by 10-fold.
• Improving MEP pathway prevents decrease in specific titres of cells grown in HDC.
• The best producing strain reached 180 ​mg ​L^−1^ bisabolene after 10 days growth in HDC.

## Graphical abstract

**Figure:**
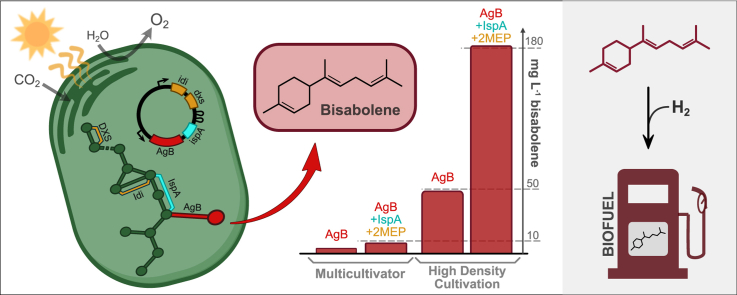

## 1 Introduction

Terpenoids are a very wide class of organic compounds with high industrial relevance. Depending on their chemical properties, these compounds can be used in the food sector as colorants, as fragrances in perfumes and cosmetics, as feedstock for chemical synthesis and potentially as biofuels and biofuel precursors (Schempp et al., 2018; Mata-Gómez et al., 2014; Rude and Schirmer, 2009). All terpenoids are produced in nature from the same 5-carbon-atom building blocks: dimethylallyl-pyrophosphate (DMAPP) and isopentenyl-pyrophosphate (IPP). Consecutive prenyl condensations of these two C5 units, catalysed by prenyl transferases, yield hydrocarbons with different carbon-chain lengths, which can then be further decorated with other functional groups (Pattanaik and Lindberg, 2015).

**Fig. 1:**
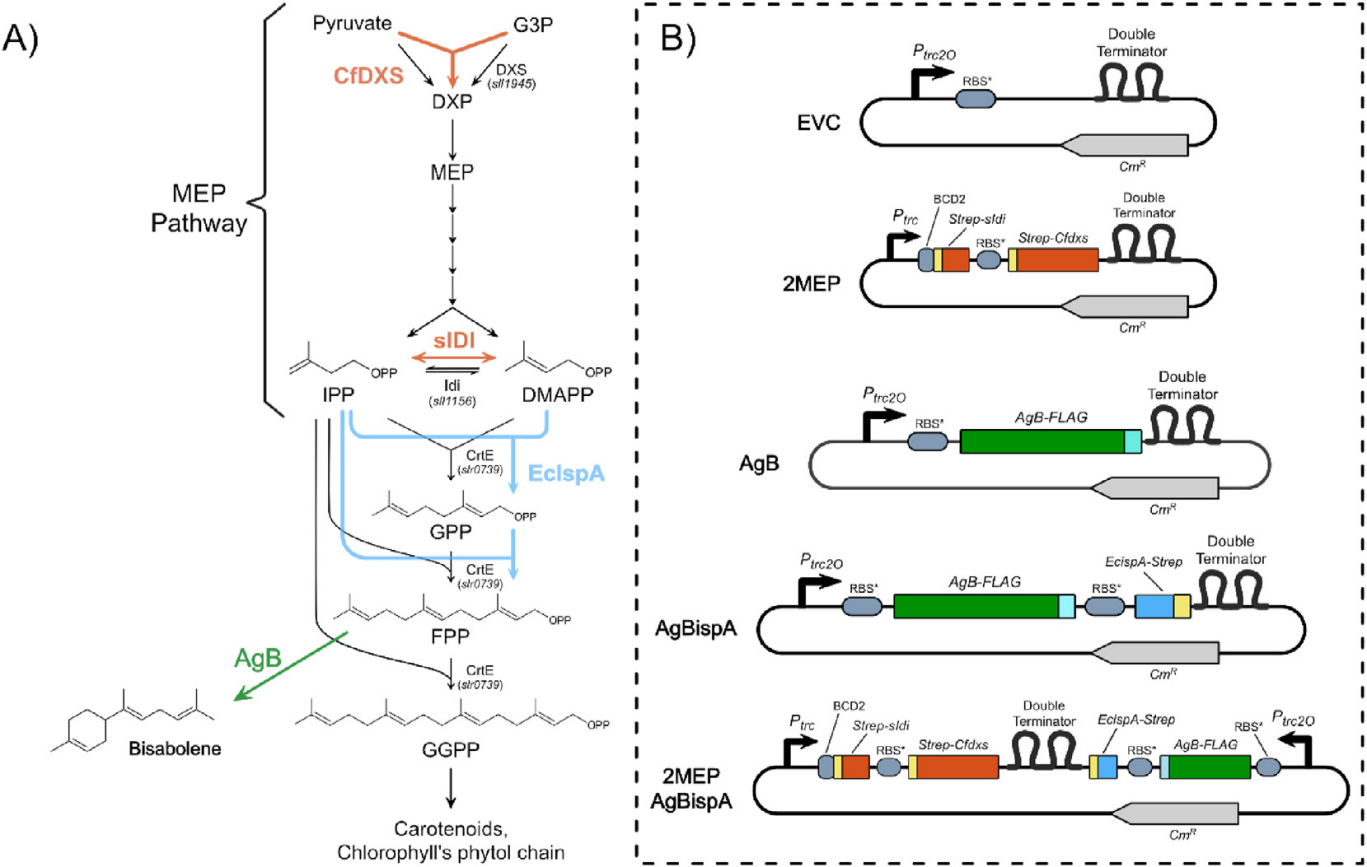
Schematic representations of: A) the native MEP pathway (in black) and the metabolic engineering strategies adopted in this study for bisabolene production (green) and carbon partitioning towards terpenoid biosynthesis (orange and blue); and B) the synthetic devices designed and built in this study. Abbreviations: G3P - glyceraldehyde-3-phosphate; DXP - 1-Deoxy-D-xylulose-5-phosphate; MEP - 2-C-methylerythritol 4-phosphate; IPP - isopentenylpyrophosphate; DMAPP - dimethylallyl-pyrophosphate; GPP - geranyl-pyrophosphate; FPP - farnesyl-pyrophosphate; GGPP - geranylgeranyl-pyrophosphate; DXS - native DXP synthase; CfDXS - DXP synthase, from *Coleus forskohlii*; sIDI - IPP/DMAPP isomerase, from *Synechocystis* sp. PCC 6803; EcIspA - FPP synthase, from *Escherichia coli*; AgB - bisabolene synthase, from *Abies grandies*; CrtE - native polyprenyl synthase). (For interpretation of the references to color in this figure legend, the reader is referred to the Web version of this article.)

Cyanobacteria are capable of producing terpenoids via the methylerythritol 4-phosphate (MEP) pathway, which relies on glyceraldehyde-3-phosphate (G3P) and pyruvate, both derived from photosynthesis (Fig. 1). These photosynthetic microorganisms produce a myriad of terpenoids that play different important roles in the physiology of the cell, such as in light harvesting as accessory pigments in the photosystems (*e.g.* β-carotene), membrane fluidity (*e.g.* hopanoids) or protection against high light and oxidative stress (*e.g.* orange carotenoid proteins) (Zakar et al., 2016; Bao et al., 2017; Rezanka et al., 2010). Given their ability to produce terpenoids from intermediates that come from photosynthesis, cyanobacteria have been subject of study during the last decade as potential solar-powered cell factories for conversion of atmospheric carbon dioxide into terpene-based compounds. Several approaches resulted in cyanobacterial chassis capable of producing a different range of compounds including isoprene (Lindberg et al., 2010; Gao et al., 2016), β-phellandrene (Formighieri and Melis, 2016) and limonene (Davies et al., 2014). However, with the exception of isoprene in the study by Gao et al. (2016), reported titres have been low.

(*E*)-α-bisabolene (C_15_H_24_) is a hydrophobic monocyclic terpenoid and a member of the sesquiterpenoid family. It is a constituent of the oleoresin of several different plants, including the fir *Abies grandis* (Bohlmann et al., 1998). The biosynthesis of bisabolene involves three isoprene units, where one molecule of DMAPP is first condensed with one molecule of IPP by the enzyme geranyl-pyrophosphate synthase (GPPS), forming geranyl-pyrophosphate (GPP); and then, a second enzyme, farnesyl-pyrophosphate synthase (FPPS), catalyses the addition of a second IPP unit to GPP, originating farnesyl-pyrophosphate (FPP). FPP is the final precursor for bisabolene biosynthesis, and its conversion into bisabolene is catalysed by the enzyme bisabolene synthase (Fig. 1) (McAndrew et al., 2011). This particular 15-carbon-atom terpenoid is of great relevance to the industry. Bisabolene is commonly used as component of perfumes and as precursor in chemical synthesis (Davies et al., 2014; Vyvyan et al., 2004). Besides their currently established applications, bisabolene was also shown to be suitable for the synthesis of biofuels both for land and air transportation (Peralta-Yahya et al., 2012; Baral et al., 2019). Peralta-Yahya et al. (2012) demonstrated that, upon chemical hydrogenation into bisabolane, this terpenoid presents similar fuel properties as those of D2 diesel. Furthermore, through heterologous expression of a codon-optimized version of bisabolene synthase from *A. grandis* (AgB) in heterotrophic microorganisms, together with an optimized version of the entire mevalonate pathway from *Saccharomyces cerevisiae*, they achieved titres as high as 1 ​g ​L^−1^ of (*E*)-α-bisabolene (Peralta-Yahya et al., 2012). In another study, done by Kim et al. (2017), bisabolene production was addressed in *Escherichia coli* using a quorum sensing system for autonomous control of the metabolic state of the cells (Kim et al., 2017). The authors used the well studied LuxI/R system from *Vibrio fischeri* as sensor to regulate the expression of bisabolene synthase and the best performing strain achieved a titre of 1.1 ​g ​L^−1^ of bisabolene without any external inducers (Kim et al., 2017). Davies et al. (2014) expressed the same bisabolene synthase in the cyanobacterium *Synechococcus* sp. PCC 7002 and demonstrated the feasibility of bisabolene production in cyanobacteria (Davies et al., 2014). Later, in a study done by Sebesta and Peebles (2020), bisabolene biosynthesis was also proven to be possible in *Synechocystis* sp. PCC 6803^18^. The authors expressed several variants of the bisabolene synthase with different codon usage sequences and ribosome binding sites (RBSs) and tested the best strain in a photobioreactor exposed to simulated outdoors light conditions. However, the titres of photosynthetically produced bisabolene are still too small to be suitable for industrial applications.

Here, we report the metabolic engineering of the native terpenoid biosynthesis pathway as means to increase the production of bisabolene in the model cyanobacterium *Synechocystis* sp. PCC 6803 (hereafter *Synechocystis*). Bisabolene-producing strains were generated through heterologous expression of a codon-optimized bisabolene synthase from *A. grandis* together with other key enzymes of the native terpenoid metabolism in order to increase carbon flow towards terpenoid biosynthesis. We also addressed the performance of the resulting bisabolene-producing strains in cultivation systems that promote growth to higher cell densities, as this strategy was proven fruitful for sesquiterpenoid biosynthesis in *Synechocystis* (Dienst et al., 2020).

## 2 Materials and methods

### 2.1 Strains and growth conditions

*Escherichia coli* strain DH5α Z1 (Expressys) was used for subcloning. Cultivation was performed at 37 ​°C in liquid LB medium supplemented with 35 ​μg ​mL^−1^ chloramphenicol. Plasmid vectors were transferred to *Synechocystis* sp. PCC 6803 wild-type strain by three-parental mating (Heidorn et al., 2011), using the *E. coli* HB101 strain harbouring the pRL443 conjugative plasmid.

*Synechocystis* cultures were maintained at OD_750_ ​< ​1 in BG11 supplemented with 20 ​μg ​mL^−1^ chloramphenicol, in 100 ​mL Erlenmeyer flasks, at 30 ​°C and 25 ​μmol photons m^−2^ s^−1^.

### 2.2 Plasmid assembly

The sequence for the bisabolene synthase from *Abies grandis* (GenBank accession number AAC24192.1), was codon optimized using the Gene Designer (DNA2.0) software, with a FLAG-tag appended at the C-terminal end for detection of the protein, and synthesized by GenScript (Piscataway, NJ, USA). The gene was inserted into the pEEC1 vector (Englund et al., 2018), using restriction enzymes *Xba*I and *Sac*II, generating the plasmid pAgB (Fig. 1).

The farnesyl-pyrophosphate synthase gene (ispA; GenBank accession number BAE76201) was amplified from *E. coli* by PCR using the primers ispA_Bgl_F (GCGCGCAGATCTTTTATTACGCTGGATGAT) and ispA_Nde_R (GCGCGCCATATGGACTTTCCGCAGCAACTC) (Table S1). The resulting fragment was purified using the GeneJET PCR Purification Kit (ThermoScientific), digested with *Bgl*II and *Nde*I and cloned in the pEEC1 vector, together with and downstream of the *AgB* gene, as an operon. The resulting vector was termed pAgBispA (Fig. 1).

The p2MEP-AgBispA construct (Fig. 1) was obtained by PCR amplification of the *AgB::ispA* operon from pAgBispA using the primers P-Ptrc2O–AgB-IpsA_F (TTCACTGCAGCTTACGTGCCCGATCAACTC) and IspA-SpeI_Rev (CGTTCACCGACAAACAACAG) (Table S1). The PCR product was purified using the GeneJET PCR Purification Kit (ThermoScientific), cut with *Pst*I and *Spe*I and ligated with the pEEC7 plasmid (Englund et al., 2018), previously cut with *Pst*I and *Xba*I and dephosphorylated with alkaline phosphatase.

All enzymatic digestions were performed with FastDigest enzymes from ThermoScientific, according to their specifications. Ligation reactions were performed using the Quick Ligation kit from New England Biolabs (NEB). PCR amplifications were performed using the Phusion High Fidelity DNA polymerase kit (ThermoScientific). The full sequence of the plasmids generated in this study are provided in the Supplementary files.

### 2.3 Bisabolene production in MC1000 multicultivators

The bisabolene production experiments were carried out using a similar setup as described in our previous study on production of sesquiterpenoids in *Synechocystis* (Dienst et al., 2020). *Synechocystis* cultures were inoculated at OD_750_ ​= ​0.2 into 60 ​mL BG11 supplemented with 50 ​mM sodium bicarbonate, 50 ​mM HEPES buffer (pH adjusted to 8.0) and 20 ​μg ​mL^−1^ chloramphenicol. An organic layer was set up to trap the bisabolene by adding 5% (v/v) dodecane (reagent grade, >99%; Honeywell) to the cultures. Each strain was grown in triplicates for 12 days in MC-1000 photobioreactors (Photon Systems Instruments) at 30 ​°C and an initial light intensity of 50 ​μmol photons m^−2^ s^−1^. It has been shown previously that this constitutes a simple way to capture sesquiterpenoids from cyanobacteria, as very low amounts of the sesquiterpenoid products are retained inside the cells or detected in the culture media (Davies et al., 2014; Dienst et al., 2020). Growth was monitored by daily OD_750_ measurements and 12 ​mL fresh medium was supplemented at days 5 and 10. In order to keep the cultures in the linear growth phase, light intensity was increased to 100 and to 150 ​μmol photons m^−2^ s^−1^ at days 7 and 10, respectively.

For pigments and protein extractions, 6 ​mL culture were collected at days 5 and 10 and centrifuged at 2700×*g* for 10 ​min. The supernatant was removed, the cells were washed with Phosphate Buffered Saline (PBS) and, after a second centrifugation step, the cells were resuspended in one volume of PBS. Two aliquots of 500 ​μL were transferred to microcentrifuge tubes, centrifuged at 18,000×*g* and the pellets were stored at −80 ​°C. The remaining 5 ​mL of cell suspension were centrifuged 10 ​min at 2700×*g*, resuspended in 1 ​mL PBS and transferred to screw-cap-tubes. The cell suspensions were then centrifuged 2 ​min at 18,000×*g*, the supernatant was discarded and the cells were stored at −80 ​°C.

For quantification of the bisabolene present in the organic layer, 200 ​μL of dodecane were sampled at days 0, 1, 3, 5, 7, 10 and 12 and transferred to GC vials (VWR). The vials were stored at −20 ​°C until further analysis by gas chromatography (GC).

### 2.4 Bisabolene production in high density cultivation system

The bisabolene production experiments were also performed in high density cultivation (HDC) systems using the HDC 6.10 started kit (Celldeg). The experiments were carried out following the procedure described by Dienst et al. (2020) (Dienst et al., 2020). In brief, seed-cultures of the bisabolene-producing *Synechocystis* strains were grown in BG11 medium supplemented with 20 ​μg ​mL^−1^ chloramphenicol and centrifuged for 5 ​min at 2700×*g*. The cell pellets were resuspended in CD medium containing chloramphenicol (see full formulation in https://www.protocols.io/view/cd-media-for-high-density-cultivation-of-synechocy-2bxgapn) to an OD_750_ of 0.35 and 8 ​mL of culture were transferred to HDC cultivation vessels. An overlay of 2 ​mL of dodecane was added to each culture. The experiment was performed in quadruplicates. Cultivation was performed at 30 ​°C under constant illumination with fluorescent white light in a Sanyo ‘Versatile Environmental Test Chamber’ (Sanyo) without humidifier. The light intensity was programmed as follows: 250 ​μmol photons m^−2^ ​s^−1^ (0–24 ​h), 490 ​μmol photons m^−2^ ​s^−1^ (24–48 ​h), 750 ​μmol photons m^−2^ ​s^−1^ (48–240 ​h). Cultures were constantly shaken at 320 ​rpm on IKA KS 130 basic orbital shakers (ø ​= ​4 ​mm). Growth was monitored by OD_750_ measurements at days 0, 2, 4 and 10. On days 4 and 10, additional 200 ​μL of culture were sampled for protein extraction and detection by Western blotting. These samples were washed once with PBS and the pellets were kept at −80 ​°C until further processing. For bisabolene quantification, 200 ​μL dodecane overlay were sampled on days 0, 2, 4 and 10 and transferred to GC vials. The samples were stored at −20 ​°C until further analysis by GC.

### 2.5 Bisabolene quantification

A calibration curve was performed using standard solutions of bisabolene (mixture of isomers; Alfa Aesar) in dodecane with concentrations of 0, 50, 100, 200, 400 and 800 ​μg ​mL^−1^. As internal standard, β-caryophyllene (>80%, FCC, FG; Sigma-Aldrich) was added to the standard solutions, as well as all the samples, to a final concentration of 250 ​μg ​mL^−1^. Bisabolene was detected by gas chromatography (PerkinElmer Gas Chromatograph Clarus 580) coupled with flame ionization detector (FID), using an Elite-WAX capillary column (30 ​m ​× ​0.25 mm ID x 0.25 ​μm film; PerkinElmer). The chromatography was performed according to the following conditions: inlet and FID at 250 ​°C, carrier gas (N_2_) at 50 ​mL ​min^−1^. The oven was set up with the following program: 100 ​°C for 1 ​min, 5 ​°C min^−1^ up to 160 ​°C, 10 ​°C min^−1^ up to 240 ​°C, 240 ​°C for 5 ​min. The compounds β-caryophyllene and (*E*)-α-bisabolene were detected on the GC run at 7.7 and 10.8 ​min, respectively.

### 2.6 Protein detection and quantification

In order to detect and quantify the overexpressed enzymes, crude protein extracts were prepared from frozen *Synechocystis* cells as described previously (Ivleva et al., 2007), and total protein concentrations were measured using the DC protein assay (BIO-RAD). For the detection by Western Blot, 20 ​μg of total protein were mixed with 4x Laemmli Sample Buffer (BIO-RAD) and incubated at 100 ​°C for 5 ​min. For the detection of the bisabolene synthase, 40 ​μg of total protein were used instead. The samples were then separated by sodium dodecyl sulfate polyacrylamide gel electrophoresis (SDS-PAGE) using 4–15%, 12-well polyacrylamide precast gradient gels (BIO-RAD). The proteins were blotted to PVDF membranes using mini transfer packs (BIO-RAD) and identified using anti-strep-tag (Abcam, ab76949) and anti-FLAG-tag (Sigma-Aldrich, F3165) primary antibodies and HRP-conjugated secondary antibodies: rabbit anti-mouse IgG for FLAG-tag detection (Agrisera, #AS10 1114); and goat anti-rabbit IgG for Strep-tag II detection (BIO-RAD, #170–5046). Bands were detected using the Clarity ECL substrate (BIO-RAD).

### 2.7 Pigment quantification

The pigment extraction was performed in 2 technical replicates according to Zavrel et al. (2015), under low light conditions. Frozen samples from the bisabolene production experiments performed in the MC1000 multicultivators were thawed on ice, resuspended in 1 ​mL MeOH 100% (Alpha Aeser) and incubated for 1 ​h at 4 ​°C, protected from the light. The samples were then centrifuged for 10 ​min at 15,000×*g* and at 4 ​°C, and the supernatant was used for the absorption spectrum measurements in the 400–750 ​nm range. The spectra were obtained in a Varian Cary 50 BIO spectrophotometer, using methanol as blank. Chlorophyll *a* (Chl *a*) and carotenoids (Car) were quantified using the following equations:

(1) $$\left[Chl\;\text{a}\right]=12.9447\;x\;\left(Abs_{665\;nm}-\;Abs_{720\;nm}\right)\;\mu g\;{\text{mL}}^{-1}$$

(2) $$\left[Car\right]=\;\frac{1000\;x\;\left(Abs_{470\;nm}-\;Abs_{720\;nm}\right)-2.86\;x\;\left[Chl\;a\right]}{221}\;\mu g\;{\text{mL}}^{-1}$$

### 2.8 Dry cell weight determination

The dry cell weight (DCW) was assessed in 2 technical replicates of at least 3 biological replicates, for the cultivation experiments performed in the MC1000 multicultivators. At the end of the experiment (day 12), 20 ​mL of culture were collected, washed with deionized water and transferred to previously weighed aluminium trays (aluminium trays, ø ​= ​43 ​mm, Heathrow Scientific). The trays containing the samples were placed in an oven and incubated at 70 ​°C. Upon complete dryness, the samples were weighed and the DCW values were determined by subtracting the initial mass of the trays.

## 3 Results & discussion

### 3.1 Experimental design for the biosynthesis of bisabolene

In order to test the production of bisabolene in *Synechocystis*, we expressed a codon-optimized version of the bisabolene synthase (*AgB*) from *Abies grandis* lacking the N-terminal transit peptide (Bohlmann et al., 1998), with a FLAG epitope tag appended to the C-terminus of the protein for detection. The gene was cloned in a self-replicating plasmid carrying chloramphenicol resistance cassette, under control of the synthetic *trc*2O promoter (AgB, Fig. 1). To investigate if precursor availability could constitute a bottleneck in bisabolene biosynthesis in *Synechocystis*, we generated a second engineered strain where AgB is co-expressed with a farnesyl-pyrophosphate synthase (*ispA*) from *E. coli*. For that, we cloned the *ispA* gene downstream *AgB* as an operon in a self-replicating plasmid (AgBispA, Fig. 1), including a C-terminal Strep-tag in the construct for detection of the protein. Finally, to further enhance redirection of the carbon flow towards terpene biosynthesis, we created a third cyanobacterial strain that overexpresses codon-optimized versions of 1-deoxy-*D*-xylulose-5-phosphate synthase (DXS) from *C. forskohlii* and IPP/DMAPP isomerase (IDI) from *Synechocystis*, as they were previously reported as bottlenecks of the MEP pathway (Englund et al., 2018). The *AgB::ispA* operon was cloned in a convergent fashion in pEEC7, a plasmid that already contained the genes that encoding DXS and IDI, both Strep-tagged at the C-terminus, driven by the *trc* promoter and a bicistronic device (2MEP-AgBispA, Fig. 1) (Englund et al., 2018). In the study done by Englund et al. (2018) on production of isoprene in *Synechocystis*, expression of DXS and IDI was investigated using different genetic elements and, from all versions tested, the abovementioned variant was the one that performed the best (Englund et al., 2018). The generated synthetic devices were introduced in our model cyanobacterium by conjugative transformation. The resulting engineered strains were confirmed by colony PCR, and protein expression was assessed by western immunoblotting. In order to evaluate whether the effects of heterologous expression on the physiology of the cyanobacterium are not due to the presence of the self-replicating vector *per se*, we used a control cyanobacterial strain carrying the empty plasmid (EVC, Fig. 1). A second control strain expressing only the heterologous DXS and IDI (2MEP, Fig. 1) was also generated to evaluate the effect of the enhancement of the MEP pathway alone on growth and pigment content and to serve as comparison to the 2MEP-AgBispA strain. Such strain was generated by transformation with the empty pEEC7 plasmid (Englund et al., 2018). Table 1 summarizes all constructs (DNA sequences can be found in S2) and resulting *Synechocystis* strains used in this study.

**Table 1:** **-** List of synthetic devices and strains used in this study.

Construct	Expressed gene(s)	Backbone plasmid	*Synechocystis* strain
pEEC1	–	pEEC1^a^	EVC (Englund et al., 2018)
pEEC7	*Cfdxs, sIdi*	pEEC7^a^	2MEP
pAgB	*AgB*	pEEC1	AgB
pAgBispA	*AgB, ispA*	pEEC1	AgBispA
p2MEP-AgBispA	*Cfdxs, sIdi*; *AgB*, *ispA*	pEEC7	2MEP-AgBispA

a pEEC1 - RSF1010-based expression vector; pEEC7 - pEEC1-based vector expressing CfDXS and sIDI in an operon regulated by *trc* promoter and BCD2 genetic insulator; contains a cloning site in the reverse direction driven by P_*trc*_. All backbone plasmids were obtained from Englund et al., 2018) (Englund et al., 2018).

### 3.2 Bisabolene production in multicultivator systems

To address the ability of the engineered strains to produce bisabolene, as well as its impact on the physiology of the cell, AgB, AgBispA and 2MEP-AgBispA strains were cultivated in parallel with the EVC and 2MEP control strains in MC1000 multicultivator tubes. The strains were cultivated for 12 days with constant aeration and bicarbonate supplementation, and growth was assessed by periodic optical density measurements. As means to capture the produced bisabolene, an organic overlay of dodecane was added to all cultures and the concentration of the desired sesquiterpenoid was monitored at different time points of the cultivation.

**Fig. 2:**
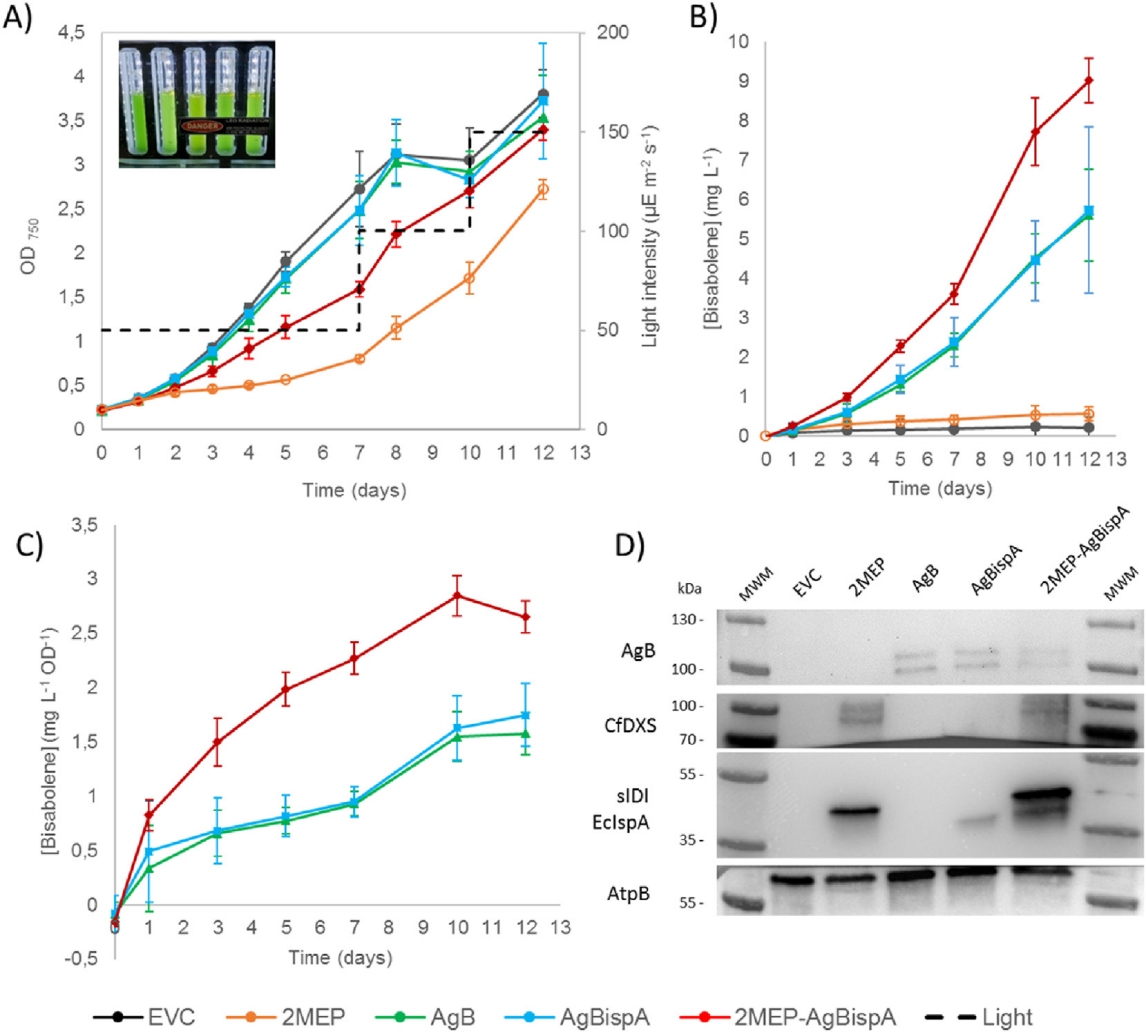
**–** Bisabolene production experiment performed in the MC1000 multicultivators. A) Growth curves of all engineered strains and light intensity profile throughout the 12 days of cultivation (inset corresponds to a picture of the cultures growing in the multicultivators). B) Total cumulative bisabolene titres, in mg L^−1^ culture, of the 5 engineered strains throughout the entire experiment. C) Specific bisabolene titres, in mg L^−1^ culture OD^−1^, of the 3 bisabolene-producing strains. D) Western immunoblot analysis of all overexpressed proteins. 20 ​μg of total protein were loaded for each strain for the detection of all Strep-tagged proteins. For the detection of FLAG-tagged AgB protein, 40 ​μg of total protein were loaded instead. The β-subunit of ATP synthase (AtpB) was used as loading control. MWM - molecular weight marker.

First, one can see from the growth patterns of EVC and AgB (Fig. 2 A) that the heterologous expression of the bisabolene synthase does not compromise the growth of our model organism. Furthermore, the expression of this enzyme is enough to complete the biosynthetic pathway of bisabolene, as bisabolene production was observed starting from the third day of the experiment and reached a titre of 5 ​mg ​L^−1^ culture after 12 days of cultivation (Fig. 2 B).

In contrast to plants and some other cyanobacteria, the *Synechocystis* genome contains only one gene (*crtE*) encoding a polyprenyl transferase, and therefore all polyprenyl precursors are most likely produced by the same enzyme. Considering that monoterpenes and sesquiterpenes are absent in many cyanobacteria, and carotenoids constitute the major class of terpenoids synthesized by these microorganisms, it is probable that the intracellular pool of GPP and FPP might be considerably low, compared to the one of GGPP (Pattanaik and Lindberg, 2015; Davies et al., 2014). Therefore, substrate limitations may impose significant constraints in bisabolene production. However, it is interesting to note that co-expression of the FPP synthase from *E. coli* (*i.e.* IspA) did not seem to improve neither total volumetric bisabolene production nor the specific yields, (Fig. 2 B and C). We also observed a negative effect of the overexpression of the two MEP enzymes alone on the growth of the strain (Fig. 2 A); such behaviour, however, was less evident for the case of the 2MEP-AgBispA strain, where AgB and IspA are expressed together with the 2 MEP enzymes (Fig. 2 A). This highlights a correlation between the growth impairment of the 2MEP strain with the lack of a proper carbon sink for the terpenoid metabolism, possibly related to an aberrant carbon flow within the pathway and consequent accumulation of intermediates with feedback effects. In the study on the MEP pathway of *E. coli* done by Volke et al. (2019), the authors demonstrated that, by increasing the expression DXS in an isoprene-producing strain with IDI overexpression, all MEP intermediates increased linearly with the abundance of DXS with the exception of MEcPP, which accumulated substantially (Volke et al., 2019). Furthermore, the authors identified an increased efflux of this intermediate as consequence of IspG saturation and MEcPP accumulation. Gao et al. (2016) also demonstrated that, when overexpressing DXS and IDI in a *Synechococcus* sp. PCC 7942 isoprene-producing strain, IspG becomes the major bottleneck in the MEP pathway (Gao et al., 2016). The same intermediate accumulation might be occurring in our 2MEP strain, which may be the reason for the observed changes in the cellular physiology. Alternatively, the growth impairment might be due to some crosstalk between the MEP pathway and other pathways and/or regulation mechanisms. In the study on limonene production in *Synechocystis* done by Lin et al. (2017), the overexpression of two enzymes of the pentose phosphate pathway led to an improvement in limonene production, indicating a possible connection between this pathway and the MEP pathway (Lin et al., 2017). One can consider that, like the pentose phosphate, other pathways may have direct interaction with terpenoid biosynthesis and therefore, interfering with the MEP pathway may impose changes to the metabolic network on a more complex level.

The overexpression of DXS and IDI together with the bisabolene and FPP synthases led to a significant increase in the bisabolene titres; the strain reached 9 ​mg ​L^−1^ culture after 12 days of cultivation (Fig. 2 B), which translates into approximately 17 ​mg ​g^−1^ DCW of bisabolene (Fig. S3). Moreover, one can see an increase in the production per cell when these two MEP enzymes are overexpressed, as 2MEP-AgBispA strain presents a specific bisabolene yield that is 1.7-fold the one from AgB or AgBispA strains (Fig. 2 C). Given the irreversible nature of the reaction catalysed by DXS, this enzyme plays an important role in the carbon partitioning for terpenoid biosynthesis. Expressing a heterologous DXS allows the increase of the carbon flow towards the MEP pathway with less interaction from native regulation mechanisms. At the end of the enzymatic cascade that composes the MEP pathway lies IDI, the enzyme responsible for the interconversion of DMAPP and IPP and thus, balancing the pool of these two terpenoid building blocks. Considering that bisabolene biosynthesis does not involve an equimolar consumption of DMAPP and IPP, overexpressing IDI helps preventing dramatic changes in the ratio between the two precursors (Gao et al., 2016; Englund et al., 2018). To confirm that this increase in production is due to the heterologous expression of the MEP bottleneck enzymes and not to different AgB protein amounts, we extracted the total protein content of all strains and performed a western immunoblotting analysis (Fig. 2 D). Similar detection signals from FLAG-tagged bisabolene synthase and the loading control (β-subunit of ATP synthase) between all bisabolene-producing strains corroborates the positive effect of overexpression of DXS and IDI on bisabolene biosynthesis.

### 3.3 Pigment content

**Fig. 3:**
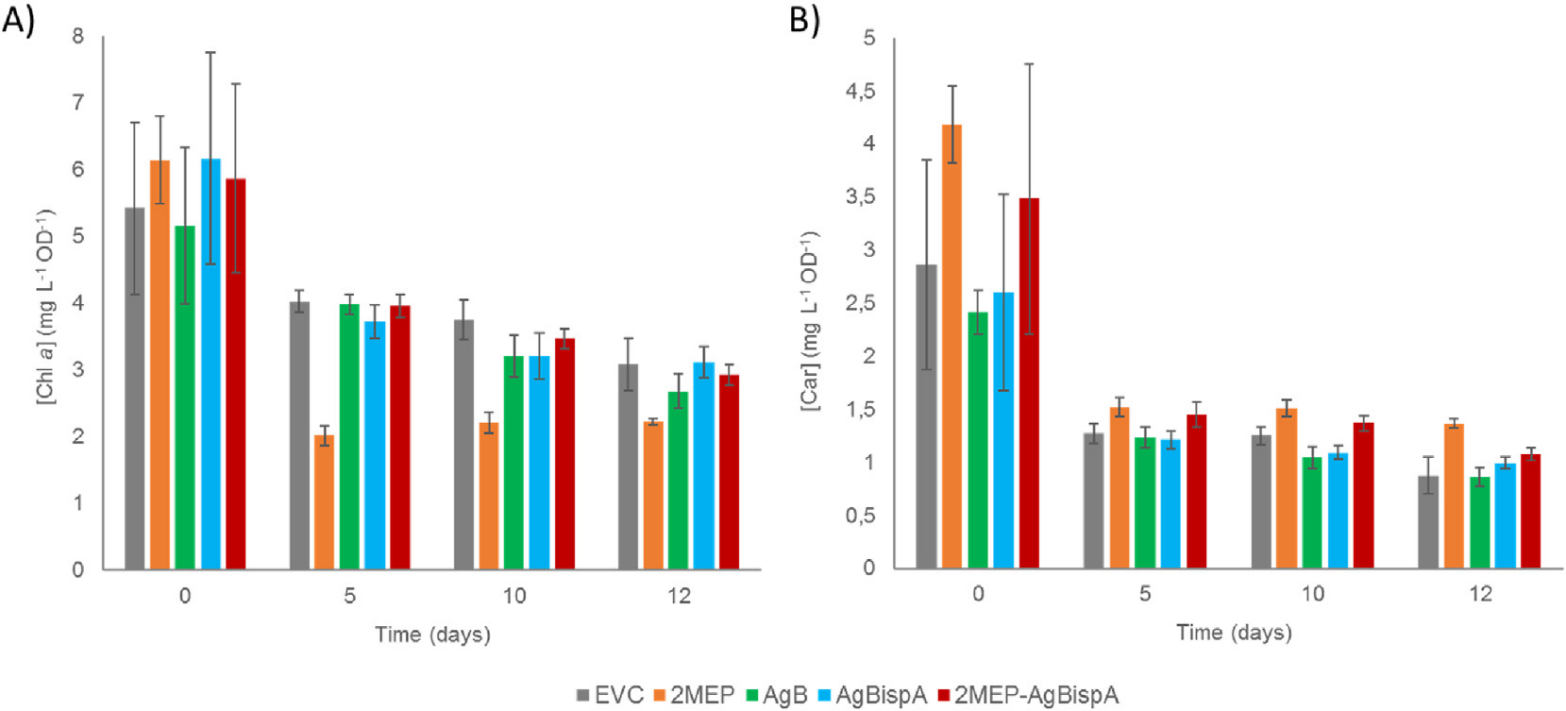
**-** Pigment content of all engineered strains at day 0 and after 5, 10 and 12 days of growth in the MC1000 multicultivators. A) Chlorophyll *a* concentration, in mg L^−1^ culture, normalized to the OD_750_. B) Total carotenoid concentration, in mg L^−1^ culture, normalized to the OD_750_.

Cyanobacteria make use of the MEP pathway to produce different terpenoids. It is thought that the majority of the carbon that is partitioned to this pathway is redirected to geranylgeranyl-pyrophosphate (GGPP) formation (Fig. 1 A), which is the precursor of all carotenoids, as well as the phytol tail of chlorophyll *a* (Pattanaik and Lindberg, 2015; Takaichi and Mochimaru, 2007). Given both bisabolene and GGPP share the same precursor (*i.e.* FPP), the introduction of the bisabolene biosynthetic pathway in *Synechocystis* could interfere with the cellular pigment content and, thus, with photosynthesis. Making a direct comparison between bisabolene and pigment production rates is not straightforward, as while the former is a cumulative value obtained throughout several days of cultivation and continuous trapping, the latter is a fixed value for a given time point, result of the dynamic relationship between synthesis and degradation of carotenoids and chlorophyll *a*. Therefore, one can only make assumptions on how bisabolene production can affect pigment formation by interpreting variations in the pigment content between strains and at same time points. We decided to analyse the chlorophyll *a* (Chl *a*) and total carotenoid (Car) content of all strains at different time points of the cultivation in the MC1000 bioreactors (Fig. 3).

We observed a relatively large variation in the pigment content of the cultures at the beginning of the experiment, which is related to possible differences on the growth of the pre-cultures. Nevertheless, the pigment content at later time points becomes uniform within biological replicates, possibly due to their acclimation to the new growth conditions. Another general observation is a tendency for both Chl *a* and Car concentrations to decrease over time (Fig. 3), which might be due to the transition of the cultures throughout different growth stages.

Looking at the pigment content of the 2MEP strain, one can see a clear decrease in the Chl *a* concentration in all time points, compared to the EVC strain (Fig. 3 A). One would not expect a decrease in the Chl *a* content when enhancing the MEP pathway, as its biosynthesis is dependent on this pathway. On one hand, the improvement of the MEP pathway might favour carotenogenesis, leading to a decrease in the carbon flow towards the phytol chain, necessary for Chl *a* assembly, which would result in a lesser ability to harvest light for water splitting. On the other hand, the growth impairment observed might not be consequence of changes in Chl *a* content, but rather its cause. Opposite to what we observed for Chl *a*, the carotenoid content is significantly increased in the 2MEP strain, and its concentration does not decrease as much over time as those of EVC strain (Fig. 3 B). Nevertheless, no significant changes were observed on neither Chl *a* nor Car content when AgB is heterologously expressed in *Synechocystis*. The same pattern is observed for the AgBispA strain. This demonstrates that, despite being competitive pathways, the introduction of this non-native terpenoid pathway did not exhaust the cellular pool of terpenoid precursors. When AgB and IspA are expressed in the 2MEP background (*i.e.* 2MEP-AgBispA strain), we did not observe any variations in the Chl *a* concentration, compared to the EVC strain (Fig. 3 A). Additionally, Car content is slightly higher than the one from EVC, presenting a pattern similar to the 2MEP strain (Fig. 3 B). These results further indicate that the bisabolene synthase does not constitute a metabolic burden to the cells. However, the Car concentration decreases more with time, when compared with the 2MEP control strain, suggesting some delay in the biosynthesis of this sesquiterpenoid. Considering the relatively slow kinetics of the terpene synthases, it might also be that the bisabolene synthase is not a strong carbon sink and thus does not create significant fluctuations on the FPP levels in the cell (Davies et al., 2014; McAndrew et al., 2011). Other factors, such as light stress, could also contribute to fluctuations in pigment content or growth, especially at lower optical densities. However, such factors do not seem to be the major contributions to the differences observed between the strains, as the phenotype the 2MEP strain presented is reverted upon introduction of a carbon sink. In light of these results, AgB seems to be the dominant bottleneck in the biosynthesis of bisabolene and therefore one should consider improving the expression of this enzyme as means to increase catalysis. In many studies on terpenoid biosynthesis in cyanobacteria, often the major significant improvement in the titres relates to an increase in the expression of the terpene synthase (Gao et al., 2016; Englund et al., 2018; Wang et al., 2016). The studies done by Formighieri and Melis on β-phellandrene constitute good examples of how much the expression of the terpene synthase affects the terpenoid titres (Formighieri and Melis, 2014, 2015). In these studies, the authors exploited the fact that *Synechocystis* expresses phycocyanin in high amounts to increase the expression levels of β-phellandrene synthase. First, they replaced the *cpc* operon by the gene encoding the terpene synthase, which led to an increase in the terpenoid titre by 22-fold (Formighieri and Melis, 2014). Later, instead of replacing the whole operon, the authors created a fusion protein of the β-phellandrene synthase with the β-subunit of phycocyanin and placed the resulting gene under regulation of the native cpc operon. As result, the terpene synthase levels reached 20% of the total protein content, and β-phellandrene titres increased further by 12-fold (Formighieri and Melis, 2015).

### 3.4 Bisabolene production in a high density cultivation system

**Fig. 4:**
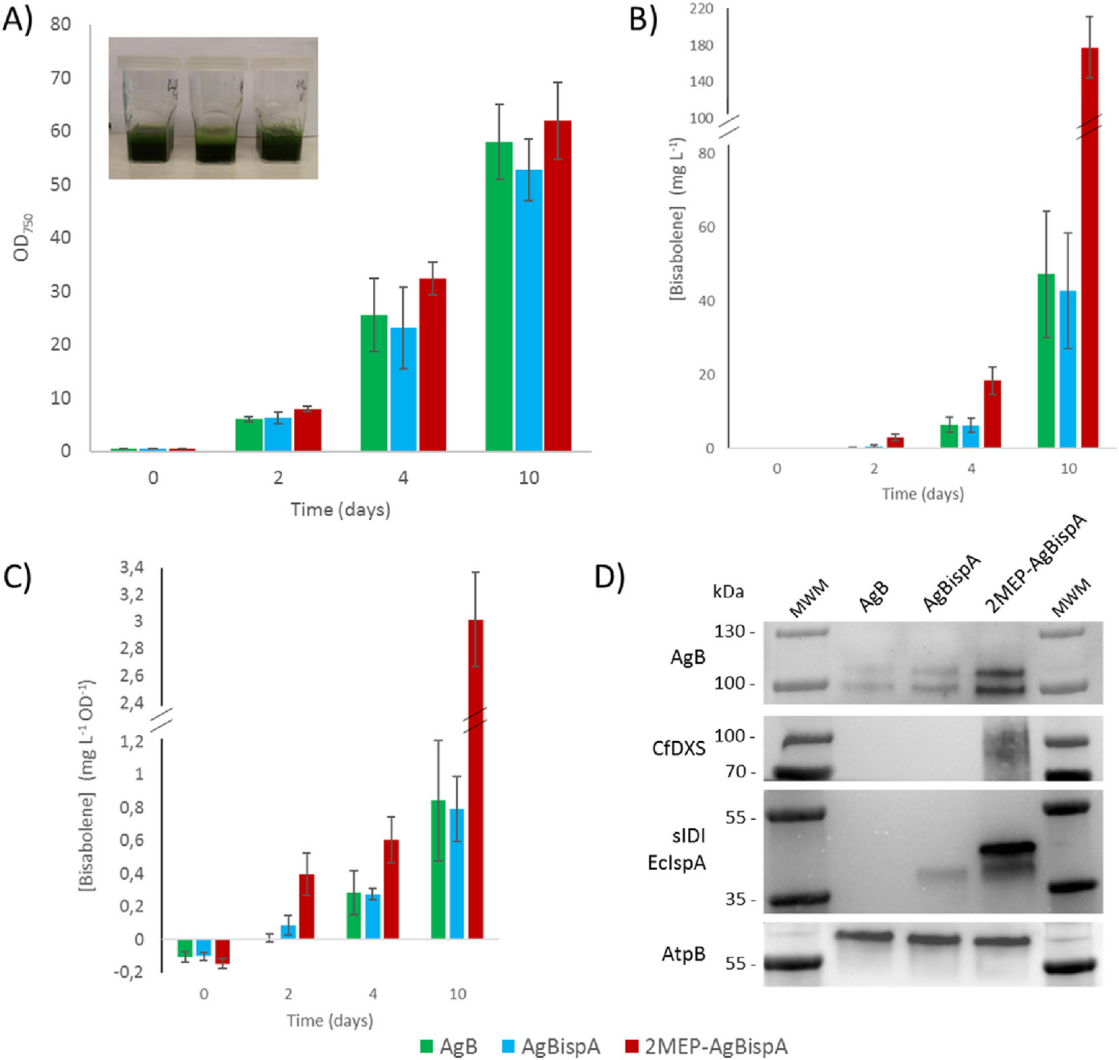
**–** Bisabolene production experiment performed in the High density cultivation (HDC) systems. A) OD_750_ of all bisabolene-producing strains at day 0 and after 2, 4 and 10 days of growth in the HDC systems (inset corresponds to a picture of the cultures growing in the HDC systems). B) Total cumulative bisabolene titres, in mg L^−1^ culture, of the 3 bisabolene-producing strains at days 0, 2, 4 and 10. C) Specific bisabolene titres, in mg L^−1^ culture OD^−1^, of the 3 bisabolene-producing strains on day 0 and after 2, 4 and 10 days of growth. D) Western immunoblot analysis of all overexpressed proteins. 20 ​μg of total protein were loaded for each strain for the detection of all Strep-tagged proteins. For the detection of FLAG-tagged AgB protein, 40 ​μg of total protein were loaded instead. The β-subunit of ATP synthase (AtpB) was used as loading control. MWM - molecular weight marker.

Previously, we have demonstrated the importance of the growth conditions on the performance of the engineered strains by studying the production of non-native sesquiterpenoids in *Synechocystis* when grown in different cultivation systems (Dienst et al., 2020). When using a high density cultivation (HDC) system, where the cultures are grown under higher light intensities, in a medium with more nutrients and constant carbon supply via CO_2_ diffusion, production was increased by one order of magnitude, compared to the standard MC1000 multicultivators. We therefore decided to try the same cultivation setup with our bisabolene-producing strains, to evaluate their performance in such conditions. We cultivated AgB, AgBispA and 2MEP-AgBispA strains in CellDeg HDC systems over 10 days in nutrient-rich mineral CD medium (Bähr et al., 2016), and growth was monitored at 0, 2, 4 and 10 days by OD_750_ measurements. A dodecane overlay was added to all cultures and bisabolene production was assessed by sampling the dodecane layer, also at 0, 2, 4 and 10 days, for GC measurements. All tested strains reached an OD_750_ of *ca.* 60 after 10 days of cultivation, and kept a healthy green aspect throughout the entire cultivation period (Fig. 4 A). Regarding bisabolene production, AgB and AgBispA reached bisabolene titres of 50 ​mg ​L^−1^ culture after 10 days of cultivation, corresponding to roughly a 9-fold increase compared to the multicultivator system (Fig. 4 B). More remarkable was the performance of the 2MEP-AgBispA in this HDC system: this strain reached a titre close to 200 ​mg ​L^−1^ culture on the 10th day of cultivation, which corresponds to an increase in production by 20-fold (Fig. 4 B). Contrarily to our previous study, where we focused on the effects of cultivation in high cell density on the production of sesquiterpenoids and therefore relied on strong inducible expression of the bisabolene synthase alone (Dienst et al., 2020), here we used a stable and constant production of bisabolene to investigate the effects of engineering the pathway on bisabolene biosynthesis. Therefore, although the final titre of our best-performing strains are similar to those reported before, the major contribution here observed comes from the overexpression of DXS and IDI - an increase of 3.6-fold compared to the expression of AgB alone. The maximum yields of the bisabolene-producing strains for both cultivation conditions are summarized in Table 2, and the maximum yields per dry cell weight content can be seen in Table S3.

**Table 2:** **-** Maximum yields of the bisabolene-producing strains using either the MC1000 multicultivators or the High Density Cultivation (HDC) system. Values in parenthesis refer to total cumulative yields.

Strain name	MC1000		HDC	
	mg L^−1^	mg L^−1^ OD^−1^	mg L^−1^	mg L^−1^ OD^−1^
AgBIS	5.1 ​± ​1.1 (5.6 ​± ​1.2)	1.43 ​± ​0.18 (1.58 ​± ​0.20)	47 ​± ​17 (50 ​± ​18)	0.8 ​± ​0.4 (0.9 ​± ​0.4)
AgBispA	5.2 ​± ​2.0 (5.7 ​± ​2.1)	1.57 ​± ​0.25 (1.75 ​± ​0.29)	43 ​± ​16 (46 ​± ​16)	0.8 ​± ​0.2 (0.8 ​± ​0.2)
2MEP-AgBispA	8.1 ​± ​0.5 (9.1 ​± ​0.6)	2.39 ​± ​0.13 (2.65 ​± ​0.15)	163 ​± ​39 (186 ​± ​34)	2.7 ​± ​0.7 (3.2 ​± ​0.3)

In our previous study, we observed a general decrease in the specific terpenoid titres of the engineered strains when grown in HDC systems, compared to the MC1000 multicultivators (Dienst et al., 2020). In these conditions, where fast growth is promoted, carbon partitioning is directed more towards biomass accumulation, *i.e.* to the primary metabolism. This behaviour was also observed for AgB and AgBispA, but not for 2MEP-AgBispA. In fact, there is no significant difference in the specific bisabolene titre of 2MEP-AgBispA between both cultivation systems (see Supplementary Fig. S4). Overexpression of a heterologous DXS can provide a way to irreversibly redirect carbon towards to MEP pathway in a way that is not subjected to native regulation and therefore counteract this tendency of the cells to prioritize primary metabolism. Immunoblot analysis also presents a slightly elevated protein expression of the bisabolene synthase in 2MEP-AgBispA, compared to the other engineered strains, which may contribute to the larger increase in the volumetric titres of this strain when comparing different cultivation setups (Fig. 4 D).

## 4 Conclusions

In this study, we aimed at altering the metabolism of *Synechocystis* towards terpenoid biosynthesis with the objective to improve bisabolene production in this model cyanobacterium. We observed that heterologous expression of an FPP synthase (*i.e.* EcIspA) did not enhance bisabolene production (neither volumetric nor specific titres). When overexpressing DXS and IDI together with AgB and EcIspA, however, the specific bisabolene titres almost doubled and a volumetric titre of 10 ​mg ​L^−1^ culture was reached after 12 days of growth in a MC1000 multicultivator setup. We also observed that, in the absence of a carbon sink, overexpression of these two MEP pathway bottleneck enzymes lead to significant growth impairment.

Furthermore, the carotenoid content was increased upon overexpression of DXS and IDI in *Synechocystis*. However, combining the bisabolene biosynthetic pathway with enhanced MEP flux did not result in any significant changes in carotenoid content. In light of these results, one can speculate that, when the MEP pathway is enhanced, the bisabolene synthase becomes the limiting step.

Cultivating the bisabolene-producing strains in high density cultivation (HDC) systems resulted in an increase in the bisabolene titres by one order of magnitude. On the other hand, the AgB and AgBispA strains presented a lower specific yield, compared with the growth in multicultivators, an effect that we had previously observed (Dienst et al., 2020). Enhancing the MEP pathway, however, allowed the 2MEP-AgBispA strain to maintain a similar specific bisabolene yield and reach volumetric titres twice as high as those for the other strains. We hypothesize that in such conditions, which promote rapid biomass formation, the cells normally prioritize the primary metabolism, leading to lower carbon partitioning towards secondary metabolites such as terpenoids, and overexpressing DXS and IDI counteracts this behaviour. After 10 days of cultivation, the best-performing strain reached a promising volumetric titre of more than 180 ​mg ​L^−1^ culture.

All together, these results highlight the relevance of improving the MEP pathway if one intends to use cyanobacteria as cell factories for terpenoid biosynthesis, especially when HDC setups are taken into consideration. Engineering of the central carbon metabolism and the co-factor balance of the cells, aiming to enhance the pool of precursors for the MEP pathway and maximize pathway flux (Englund et al., 2018), as well as implementation of mechanisms for enhanced photosynthesis, may also be beneficial for productivity in future studies. Furthermore, it seems that once the MEP pathway is improved, the bisabolene synthase becomes the bottleneck and, therefore, further improvements on both protein expression and activity are required for its application in larger scales.

## CRediT authorship contribution statement

João S Rodrigues: Conceptualization, Data curation, Investigation, Methodology, Validation, Visualization, Writing - original draft, Writing - review and editing. Pia Lindberg: Conceptualization, Data curation, Investigation, Supervision, Funding acquisition, Project administration, Writing - review and editing.

## Declaration of competing interest

The authors declare that they have no known competing financial interests or personal relationships that could have appeared to influence the work reported in this paper.

## References

[bib1] Bähr L., Wüstenberg A., Ehwald R. (2016). Two-tier vessel for photoautotrophic high-density cultures. J. Appl. phycology.

[bib2] Bao H., Melnicki M.R., Kerfeld C.A. (2017). Structure and functions of orange carotenoid protein homologs in cyanobacteria. Curr. Opin. Plant Biol..

[bib3] Baral N.R., Kavvada O., Mendez-Perez D., Mukhopadhyay A., Lee T.S., Simmons B.A., Scown C.D. (2019). Techno-economic analysis and life-cycle greenhouse gas mitigation cost of five routes to bio-jet fuel blendstocks. Energy Environ. Sci..

[bib4] Bohlmann J., Crock J., Jetter R., Croteau R. (1998). Terpenoid-based defenses in conifers: CDNA cloning, characterization, and functional expression of wound-inducible (E)-Alpha-Bisabolene synthase from grand fir (Abies grandis). Proc. Natl. Acad. Sci. U. S. A.

[bib5] Davies F.K., Work V.H., Beliaev A.S., Posewitz M.C. (2014). Engineering limonene and bisabolene production in wild type and a glycogen-deficient mutant of *Synechococcus* sp. PCC 7002. Front. Bioeng. Biotechnol..

[bib6] Dienst D., Wichmann J., Mantovani O., Rodrigues J.S., Lindberg P. (2020). High density cultivation for efficient sesquiterpenoid biosynthesis in *Synechocystis* sp. PCC 6803. Sci. Rep..

[bib7] Englund E., Shabestary K., Hudson E.P., Lindberg P. (2018). Systematic overexpression study to find target enzymes enhancing production of terpenes in *Synechocystis* PCC 6803, using isoprene as a model compound. Metab. Eng..

[bib8] Formighieri C., Melis A. (2014). Regulation of β-phellandrene synthase gene expression, recombinant protein accumulation, and monoterpene hydrocarbons production in *Synechocystis* transformants. Planta.

[bib9] Formighieri C., Melis A. (2015). A Phycocyanin·phellandrene synthase fusion enhances recombinant protein expression and β-phellandrene (monoterpene) hydrocarbons production in *Synechocystis* (cyanobacteria). Metab. Eng..

[bib10] Formighieri C., Melis A. (2016). Sustainable heterologous production of terpene hydrocarbons in cyanobacteria. Photosynth. Res..

[bib11] Gao X., Gao F., Liu D., Zhang H., Nie X., Yang C. (2016). Engineering the methylerythritol phosphate pathway in cyanobacteria for photosynthetic isoprene production from CO2. Energy Environ. Sci..

[bib12] Heidorn T., Camsund D., Huang H.-H., Lindberg P., Oliveira P., Stensjö K., Lindblad P. (2011).

[bib13] Ivleva N.B., Golden S.S., Rosato E. (2007). Protein extraction, fractionation, and purification from cyanobacteria. Circadian Rhythms: Methods and Protocols.

[bib14] Kim E.M., Woo H.M., Tian T., Yilmaz S., Javidpour P., Keasling J.D., Lee T.S. (2017). Autonomous control of metabolic state by a quorum sensing (QS)-Mediated regulator for bisabolene production in engineered *E. Coli*. Metab. Eng..

[bib15] Lin P.C., Saha R., Zhang F., Pakrasi H.B. (2017). Metabolic engineering of the pentose phosphate pathway for enhanced limonene production in the cyanobacterium *Synechocystis* sp. PCC. *Sci. Rep.*.

[bib16] Lindberg P., Park S., Melis A. (2010). Engineering a platform for photosynthetic isoprene production in cyanobacteria, using *Synechocystis* as the model organism. Metab. Eng..

[bib17] Mata-Gómez L.C., Montañez J.C., Méndez-Zavala A., Aguilar C.N. (2014). Biotechnological production of carotenoids by yeasts: an overview. Microb. Cell Factories.

[bib18] McAndrew R.P., Peralta-Yahya P.P., Degiovanni A., Pereira J.H., Hadi M.Z., Keasling J.D., Adams P.D. (2011). Structure of a three-domain sesquiterpene synthase: a prospective target for advanced biofuels production. Structure.

[bib19] Pattanaik B., Lindberg P. (2015). Terpenoids and their biosynthesis in cyanobacteria. Life.

[bib20] Peralta-Yahya P.P., Zhang F., Del Cardayre S.B., Keasling J.D. (2012). Microbial engineering for the production of advanced biofuels. Nature.

[bib21] Rezanka T., Siristova L., Melzoch K., Sigler K. (2010). Hopanoids in bacteria and cyanobacteria – their role in cellular biochemistry and physiology, analysis and occurrence. Mini-Reviews Org. Chem..

[bib22] Rude M.A., Schirmer A. (2009). New microbial fuels: a biotech perspective. Curr. Opin. Microbiol..

[bib23] Schempp F.M., Drummond L., Buchhaupt M., Schrader J. (2018). Microbial cell factories for the production of terpenoid flavor and fragrance compounds. J. Agric. Food Chem..

[bib24] Sebesta J., Peebles C.A. (2020). Improving heterologous protein expression in *Synechocystis* sp. PCC 6803 for alpha-bisabolene production. Metab. Eng. Commun..

[bib25] Takaichi S., Mochimaru M. (2007). Carotenoids and carotenogenesis in cyanobacteria: unique ketocarotenoids and carotenoid glycosides. Cell. Mol. Life Sci..

[bib26] Volke D.C., Rohwer J., Fischer R., Jennewein S. (2019). Investigation of the methylerythritol 4-phosphate pathway for microbial terpenoid production through metabolic control analysis. Microb. Cell Factories.

[bib27] Vyvyan J.R., Loitz C., Looper R.E., Mattingly C.S., Peterson E.A., Staben S.T. (2004). Synthesis of aromatic bisabolene natural products via palladium-catalyzed cross-couplings of organozine reagents. J. Org. Chem..

[bib28] Wang X., Liu W., Xin C., Zheng Y., Cheng Y., Sun S., Li R., Zhu X.-G., Dai S.Y., Rentzepis P.M. (2016). Enhanced limonene production in cyanobacteria reveals photosynthesis limitations. Proc. Natl. Acad. Sci. Unit. States Am..

[bib29] Zakar T., Laczko-Dobos H., Toth T.N., Gombos Z. (2016). Carotenoids assist in cyanobacterial photosystem II assembly and function. Front. Plant Sci..

[bib30] Zavrel T., Sinetova M.A., Červený J. (2015). Measurement of chlorophyll a and carotenoids concentration in cyanobacteria. Bio-protocol.

