# Supplementary material for: Metabolic engineering of Synechocystis sp. PCC 6803 for improved bisabolene production
Source: Metab Eng Commun. 2020 Dec 25;12:e00159. doi: 10.1016/j.mec.2020.e00159 (PMC7809396; doi:10.1016/j.mec.2020.e00159)

**Supplementary material**

**Table S1 -** List of primers used in the assembly of the synthetic devices pAgBispA and p2MEP-AgBispA. Primer overhangs in red and restriction sites underlined.

| **Primer** | **Sequence (5’ – 3’)** | **Purpose** |
| --- | --- | --- |
| ispA_Bgl_F | GCGCGCAGATCTTTTATTACGCTGGATGAT | *ispA* amplification from *E. coli* for AgBispA |
| ispA_Nde_R | GCGCGCCATATGGACTTTCCGCAGCAACTC |  |
| P-Ptrc2O-AgB-IpsA_F | TTCACTGCAGCTTACGTGCCCGATCAACTC | *AgBispA* operon amplification for 2MEP-AgBispA |
| IspA-SpeI_Rev | CGTTCACCGACAAACAACAG |  |

**S2 -** DNA sequence of the constructs designed and assembled in this study.

**A)** pAgB

## EcoRI

GAATTC

## Ptrc2O

CGAATTGTGAGCGCTCACAATTCGAACGGTTCTGGCAAATATTCTGAAATGAGCTGTTGACAATTAATCATCCGGCTCGTATAATGTGTGGAATTGTGAGCGGATAACAATTTCACACA

## Spacer

TACTCGAG

## **RBS***

TAGTGGAGGT

## XbaI

TCTAGA

## AgB

**ATG**GCCGGTGTGAGTGCCGTTAGCAAAGTGAGTTCTCTGGTGTGCGATCTGTCTAGCACTTCCGGGCTCATTCGGCGTACAGCCAACCCTCATCCAAATGTGTGGGGGTACGATCTGGTGCACTCTTTGAAATCTCCCTATATCGATTCTTCCTATCGTGAACGGGCTGAAGTGCTCGTTTCTGAGATCAAAGCCATGCTGAATCCCGCTATTACCGGCGACGGGGAATCCATGATTACCCCATCCGCCTATGATACTGCCTGGGTTGCCCGCGTTCCCGCAATTGATGGTAGCGCCCGTCCCCAATTTCCCCAAACGGTTGACTGGATTCTGAAGAACCAGTTAAAAGACGGCTCTTGGGGAATCCAATCCCACTTTCTATTGTCCGATCGGTTATTAGCCACCTTGAGCTGCGTCTTAGTATTATTGAAGTGGAACGTTGGCGATCTCCAAGTGGAACAGGGCATTGAATTTATTAAAAGTAATCTCGAGCTAGTCAAGGACGAAACGGATCAGGATAGTTTGGTGACAGATTTTGAGATTATTTTCCCCAGTTTACTGCGTGAAGCGCAGTCCCTGCGGTTGGGACTGCCGTATGACTTACCTTATATCCATTTGTTACAAACAAAACGACAAGAACGATTAGCTAAGCTGTCTCGCGAGGAAATCTACGCTGTGCCTAGTCCTCTGTTATACTCCTTAGAAGGTATTCAGGATATCGTCGAATGGGAACGTATTATGGAAGTTCAGAGTCAAGACGGAAGTTTTTTGAGCAGCCCAGCCTCCACTGCTTGTGTGTTTATGCATACCGGTGATGCCAAATGTTTAGAATTTCTCAATTCTGTGATGATCAAATTTGGCAATTTTGTGCCATGTTTATATCCAGTAGACTTGTTGGAACGGCTGTTGATTGTTGATAATATCGTCCGGTTGGGGATTTACCGTCATTTTGAGAAGGAGATTAAAGAAGCGCTGGATTATGTTTACCGCCATTGGAATGAGCGCGGGATTGGTTGGGGTCGGCTAAATCCCATTGCGGACTTAGAAACTACCGCATTGGGCTTTCGCTTGCTACGTCTGCATCGTTATAATGTGTCCCCCGCGATTTTTGATAACTTTAAAGATGCCAATGGCAAGTTTATTTGCAGCACAGGGCAGTTTAATAAAGATGTGGCGTCCATGTTAAATCTCTACCGCGCCTCTCAATTAGCCTTTCCTGGTGAAAATATTTTGGACGAAGCCAAATCTTTTGCGACGAAATACCTCCGCGAGGCGCTCGAAAAGAGCGAAACCTCCTCCGCTTGGAATAACAAACAGAATCTGTCCCAAGAAATTAAATATGCCTTAAAGACCTCCTGGCACGCAAGCGTACCTCGGGTTGAAGCTAAGCGCTATTGCCAGGTGTACCGCCCTGATTACGCCCGCATTGCCAAGTGTGTTTACAAACTCCCCTACGTCAACAACGAGAAATTCTTGGAACTCGGGAAATTAGACTTCAATATTATTCAGAGTATTCATCAAGAAGAAATGAAGAATGTAACCAGTTGGTTTCGTGACAGTGGTCTACCCTTGTTTACCTTTGCCCGAGAACGTCCCCTAGAGTTTTACTTTCTGGTGGCAGCGGGTACCTACGAACCCCAATACGCCAAGTGCCGGTTTTTGTTTACCAAAGTGGCCTGTTTACAAACCGTCCTCGATGACATGTATGATACCTATGGCACATTAGATGAATTAAAATTGTTTACCGAAGCCGTCCGTCGGTGGGACCTGAGTTTTACCGAAAATCTGCCCGATTACATGAAGTTATGCTATCAAATTTATTATGATATTGTGCACGAAGTTGCCTGGGAAGCCGAAAAAGAACAAGGCCGCGAATTGGTGAGCTTTTTTCGTAAAGGGTGGGAGGACTATCTATTGGGCTACTATGAAGAAGCTGAGTGGTTAGCCGCCGAATATGTGCCCACCTTAGATGAATATATTAAGAATGGCATCACCAGCATTGGACAACGCATTTTGTTGTTAAGCGGCGTTTTGATTATGGACGGCCAATTACTATCCCAAGAGGCTCTCGAAAAAGTCGACTACCCCGGGCGGCGTGTTCTAACTGAACTCAATTCTTTAATTTCCCGGTTAGCCGACGATACCAAAACATACAAAGCTGAAAAAGCCCGTGGCGAATTGGCCAGCAGTATTGAATGTTATATGAAAGATCACCCCGAATGTACAGAAGAAGAAGCTCTGGATCACATCTATAGCATCTTAGAACCGGCCGTCAAAGAGCTAACCCGCGAATTTTTAAAGCCAGACGATGTGCCCTTTGCGTGCAAAAAAATGCTCTTCGAAGAAACCCGTGTTACTATGGTGATTTTCAAAGATGGTGATGGTTTTGGAGTGAGCAAGCTAGAAGTTAAAGATCATATCAAGGAGTGCTTGATTGAACCTTTGCCCCTCCGGTCCGGTAGCGGAAGT

## FLAG-tag

GACTATAAGGATGATGATGACAAGGGA**TAG**

## SacII

CCGCGG

CCAGGCAT

## rrnB T1 Terminator

CAAATAAAACGAAAGGCTCAGTCGAAAGACTGGGCCTTTCGTTTTATCTGTTGTTTGTCGGTGAACGCTCTC

TACTAGAGTCACACT

## T7Te Terminator

GGCTCACCTTCGGGTGGGCCTTTCTGCG

TTTATA

## PstI

CTGCAG

## pPMQC1

TCCGGCAAAAAAGGGCAAGGTGTCACCACCCTGCCCTTTTTCTTTAAAACCGAAAAGATTACTTCGCGTTATGCAGGCTTCCTCGCTCACTGACTCGCTGCGCTCGGTCGTTCGGCTGCGGCGAGCGGTATCAGCTCACTCAAAGGCGGTAATCAATTGAGTTCTTTTACCCTCAGCCGAAATGCCTGCCGTTGCTAGACATTGCCAGCCAGTGCCCGTCACTCCCGTACTAACTGTCACGAACCCCTGCAATAACTGTCACGCCCCCCTGCAATAACTGTCACGAACCCCTGCAATAACTGTCACGCCCCCAAACCTGCAAACCCAGCAGGGGCGGGGGCTGGCGGGGTGTTGGAAAAATCCATCCATGATTATCTAAGAATAATCCACTAGGCGCGGTTATCAGCGCCCTTGTGGGGCGCTGCTGCCCTTGCCCAATATGCCCGGCCAGAGGCCGGATAGCTGGTCTATTCGCTGCGCTAGGCTACACACCGCCCCACCGCTGCGCGGCAGGGGGAAAGGCGGGCAAAGCCCGCTAAACCCCACACCAAACCCCGCAGAAATACGCTGGAGCGCTTTTAGCCGCTTTAGCGGCCTTTCCCCCTACCCGAAGGGTGGGGGCGCGTGTGCAGCCCCGCAGGGCCTGTCTCGGTCGATCATTCAGCCCGGCTCATCCTTCTGGCGTGGCGGCAGACCGAACAAGGCGCGGTCGTGGTCGCGTTCAAGGTACGCATCCATTGCCGCCATGAGCCGATCCTCCGGCCACTCGCTGCTGTTCACCTTGGCCAAAATCATGGCCCCCACCAGCACCTTGCGCCTTGTTTCGTTCTTGCGCTCTTGCTGCTGTTCCCTTGCCCGCACCCGCTGAATTTCGGCATTGATTCGCGCTCGTTGTTCTTCGAGCTTGGCCAGCCGATCCGCCGCCTTGTTGCTCCCCTTAACCATCTTGACACCCCATTGTTAATGTGCTGTCTCGTAGGCTATCATGGAGGCACAGCGGCGGCAATCCCGACCCTACTTTGTAGGGGAGGGCGCACTTACCGGTTTCTCTTCGAGAAACTGGCCTAACGGCCACCCTTCGGGCGGTGCGCTCTCCGAGGGCCATTGCATGGAGCCGAAAAGCAAAAGCAACAGCGAGGCAGCATGGCGATTTATCACCTTACGGCGAAAACCGGCAGCAGGTCGGGCGGCCAATCGGCCAGGGCCAAGGCCGACTACATCCAGCGCGAAGGCAAGTATGCCCGCGACATGGATGAAGTCTTGCACGCCGAATCCGGGCACATGCCGGAGTTCGTCGAGCGGCCCGCCGACTACTGGGATGCTGCCGACCTGTATGAACGCGCCAATGGGCGGCTGTTCAAGGAGGTCGAATTTGCCCTGCCGGTCGAGCTGACCCTCGACCAGCAGAAGGCGCTGGCGTCCGAGTTCGCCCAGCACCTGACCGGTGCCGAGCGCCTGCCGTATACGCTGGCCATCCATGCCGGTGGCGGCGAGAACCCGCACTGCCACCTGATGATCTCCGAGCGGATCAATGACGGCATCGAGCGGCCCGCCGCTCAGTGGTTCAAGCGGTACAACGGCAAGACCCCGGAGAAGGGCGGGGCACAGAAGACCGAAGCGCTCAAGCCCAAGGCATGGCTTGAGCAGACCCGCGAGGCATGGGCCGACCATGCCAACCGGGCATTAGAGCGGGCTGGCCACGACGCCCGCATTGACCACAGAACACTTGAGGCGCAGGGCATCGAGCGCCTGCCCGGTGTTCACCTGGGGCCGAACGTGGTGGAGATGGAAGGCCGGGGCATCCGCACCGACCGGGCAGACGTGGCCCTGAACATCGACACCGCCAACGCCCAGATCATCGACTTACAGGAATACCGGGAGGCAATAGACCATGAACGCAATCGACAGAGTGAAGAAATCCAGAGGCATCAACGAGTTAGCGGAGCAGATCGAACCGCTGGCCCAGAGCATGGCGACACTGGCCGACGAAGCCCGGCAGGTCATGAGCCAGACCCAGCAGGCCAGCGAGGCGCAGGCGGCGGAGTGGCTGAAAGCCCAGCGCCAGACAGGGGCGGCATGGGTGGAGCTGGCCAAAGAGTTGCGGGAGGTAGCCGCCGAGGTGAGCAGCGCCGCGCAGAGCGCCCGGAGCGCGTCGCGGGGGTGGCACTGGAAGCTATGGCTAACCGTGATGCTGGCTTCCATGATGCCTACGGTGGTGCTGCTGATCGCATCGTTGCTCTTGCTCGACCTGACGCCACTGACAACCGAGGACGGCTCGATCTGGCTGCGCTTGGTGGCCCGATGAAGAACGACAGGACTTTGCAGGCCATAGGCCGACAGCTCAAGGCCATGGGCTGTGAGCGCTTCGATATCGGCGTCAGGGACGCCACCACCGGCCAGATGATGAACCGGGAATGGTCAGCCGCCGAAGTGCTCCAGAACACGCCATGGCTCAAGCGGATGAATGCCCAGGGCAATGACGTGTATATCAGGCCCGCCGAGCAGGAGCGGCATGGTCTGGTGCTGGTGGACGACCTCAGCGAGTTTGACCTGGATGACATGAAAGCCGAGGGCCGGGAGCCTGCCCTGGTAGTGGAAACCAGCCCGAAGAACTATCAGGCATGGGTCAAGGTGGCCGACGCCGCAGGCGGTGAACTTCGGGGGCAGATTGCCCGGACGCTGGCCAGCGAGTACGACGCCGACCCGGCCAGCGCCGACAGCCGCCACTATGGCCGCTTGGCGGGCTTCACCAACCGCAAGGACAAGCACACCACCCGCGCCGGTTATCAGCCGTGGGTGCTGCTGCGTGAATCCAAGGGCAAGACCGCCACCGCTGGCCCGGCGCTGGTGCAGCAGGCTGGCCAGCAGATCGAGCAGGCCCAGCGGCAGCAGGAGAAGGCCCGCAGGCTGGCCAGCCTCGAACTGCCCGAGCGGCAGCTTAGCCGCCACCGGCGCACGGCGCTGGACGAGTACCGCAGCGAGATGGCCGGGCTGGTCAAGCGCTTCGGTGATGACCTCAGCAAGTGCGACTTTATCGCCGCGCAGAAGCTGGCCAGCCGGGGCCGCAGTGCCGAGGAAATCGGCAAGGCCATGGCCGAGGCCAGCCCAGCGCTGGCAGAGCGCAAGCCCGGCCACGAAGCGGATTACATCGAGCGCACCGTCAGCAAGGTCATGGGTCTGCCCAGCGTCCAGCTTGCGCGGGCCGAGCTGGCACGGGCACCGGCACCCCGCCAGCGAGGCATGGACAGGGGCGGGCCAGATTTCAGCATGTAGTGCTTGCGTTGGTACTCACGCCTGTTATACTATGAGTACTCACGCACAGAAGGGGGTTTTATGGAATACGAAAAAAGCGCTTCAGGGTCGGTCTACCTGATCAAAAGTGACAAGGGCTATTGGTTGCCCGGTGGCTTTGGTTATACGTCAAACAAGGCCGAGGCTGGCCGCTTTTCAGTCGCTGATATGGCCAGCCTTAACCTTGACGGCTGCACCTTGTCCTTGTTCCGCGAAGACAAGCCTTTCGGCCCCGGCAAGTTTCTCGGTGACTGATATGAAAGACCAAAAGGACAAGCAGACCGGCGACCTGCTGGCCAGCCCTGACGCTGTACGCCAAGCGCGATATGCCGAGCGCATGAAGGCCAAAGGGATGCGTCAGCGCAAGTTCTGGCTGACCGACGACGAATACGAGGCGCTGCGCGAGTGCCTGGAAGAACTCAGAGCGGCGCAGGGCGGGGGTAGTGACCCCGCCAGCGCCTAACCACCAACTGCCTGCAAAGGAGGCAATCAATGGCTACCCATAAGCCTATCAATATTCTGGAGGCGTTCGCAGCAGCGCCGCCACCGCTGGACTACGTTTTGCCCAACATGGTGGCCGGTACGGTCGGGGCGCTGGTGTCGCCCGGTGGTGCCGGTAAATCCATGCTGGCCCTGCAACTGGCCGCACAGATTGCAGGCGGGCCGGATCTGCTGGAGGTGGGCGAACTGCCCACCGGCCCGGTGATCTACCTGCCCGCCGAAGACCCGCCCACCGCCATTCATCACCGCCTGCACGCCCTTGGGGCGCACCTCAGCGCCGAGGAACGGCAAGCCGTGGCTGACGGCCTGCTGATCCAGCCGCTGATCGGCAGCCTGCCCAACATCATGGCCCCGGAGTGGTTCGACGGCCTCAAGCGCGCCGCCGAGGGCCGCCGCCTGATGGTGCTGGACACGCTGCGCCGGTTCCACATCGAGGAAGAAAACGCCAGCGGCCCCATGGCCCAGGTCATCGGTCGCATGGAGGCCATCGCCGCCGATACCGGGTGCTCTATCGTGTTCCTGCACCATGCCAGCAAGGGCGCGGCCATGATGGGCGCAGGCGACCAGCAGCAGGCCAGCCGGGGCAGCTCGGTACTGGTCGATAACATCCGCTGGCAGTCCTACCTGTCGAGCATGACCAGCGCCGAGGCCGAGGAATGGGGTGTGGACGACGACCAGCGCCGGTTCTTCGTCCGCTTCGGTGTGAGCAAGGCCAACTATGGCGCACCGTTCGCTGATCGGTGGTTCAGGCGGCATGACGGCGGGGTGCTCAAGCCCGCCGTGCTGGAGAGGCAGCGCAAGAGCAAGGGGGTGCCCCGTGGTGAAGCCTAAGAACAAGCACAGCCTCAGCCACGTCCGGCACGACCCGGCGCACTGTCTGGCCCCCGGCCTGTTCCGTGCCCTCAAGCGGGGCGAGCGCAAGCGCAGCAAGCTGGACGTGACGTATGACTACGGCGACGGCAAGCGGATCGAGTTCAGCGGCCCGGAGCCGCTGGGCGCTGATGATCTGCGCATCCTGCAAGGGCTGGTGGCCATGGCTGGGCCTAATGGCCTAGTGCTTGGCCCGGAACCCAAGACCGAAGGCGGACGGCAGCTCCGGCTGTTCCTGGAACCCAAGTGGGAGGCCGTCACCGCTGATGCCATGGTGGTCAAAGGTAGCTATCGGGCGCTGGCAAAGGAAATCGGGGCAGAGGTCGATAGTGGTGGGGCGCTCAAGCACATACAGGACTGCATCGAGCGCCTTTGGAAGGTATCCATCATCGCCCAGAATGGCCGCAAGCGGCAGGGGTTTCGGCTGCTGTCGGAGTACGCCAGCGACGAGGCGGACGGGCGCCTGTACGTGGCCCTGAACCCCTTGATCGCGCAGGCCGTCATGGGTGGCGGCCAGCATGTGCGCATCAGCATGGACGAGGTGCGGGCGCTGGACAGCGAAACCGCCCGCCTGCTGCACCAGCGGCTGTGTGGCTGGATCGACCCCGGCAAAACCGGCAAGGCTTCCATAGATACCTTGTGCGGCTATGTCTGGCCGTCAGAGGCCAGTGGTTCGACCATGCGCAAGCGCCGCCAGCGGGTGCGCGAGGCGTTGCCGGAGCTGGTCGCGCTGGGCTGGACGGTAACCGAGTTCGCGGCGGGCAAGTACGACATCACCCGGCCCAAGGCGGCAGGCTGACCCCCCCCACTCTATTGTAAACAAGACATTTTTATCTTTTATATTCAATGGCTTATTTTCCTGCTAATCAGCTCGAGGCTTGGATTCTCACCAATAAAAAACGCCCGGCGGCAACCGAGCGTTCTGAACAAATCCAGATGGAGTTCTGAGGTCATTACTGGATCTATCAACAGGAGTCCAAGCGAGCTCGATATCAAATTACGCCCCGCCCTGCCACTCATCGCAGTACTGTTGTAATTCATTAAGCATTCTGCCGACATGGAAGCCATCACAAACGGCATGATGAACCTGAATCGCCAGCGGCATCAGCACCTTGTCGCCTTGCGTATAATATTTGCCCATGGTGAAAACGGGGGCGAAGAAGTTGTCCATATTGGCCACGTTTAAATCAAAACTGGTGAAACTCACCCAGGGATTGGCTGAGACGAAAAACATATTCTCAATAAACCCTTTAGGGAAATAGGCCAGGTTTTCACCGTAACACGCCACATCTTGCGAATATATGTGTAGAAACTGCCGGAAATCGTCGTGGTATTCACTCCAGAGCGATGAAAACGTTTCAGTTTGCTCATGGAAAACGGTGTAACAAGGGTGAACACTATCCCATATCACCAGCTCACCGTCTTTCATTGCCATACGAAATTCCGGATGAGCATTCATCAGGCGGGCAAGAATGTGAATAAAGGCCGGATAAAACTTGTGCTTATTTTTCTTTACGGTCTTTAAAAAGGCCGTAATATCCAGCTGAACGGTCTGGTTATAGGTACATTGAGCAACTGACTGAAATGCCTCAAAATGTTCTTTACGATGCCATTGGGATATATCAACGGTGGTATATCCAGTGATTTTTTTCTCCATTTTAGCTTCCTTAGCTCCTGAAAATCTCGATAACTCAAAAAATACGCCCGGTAGTGATCTTATTTCATTATGGTGAAAGTTGGAACCTCTTACGTGCCCGATCAACTCGAGTGCCACCTGACGTCTAAGAAACCATTATTATCATGACATTAACCTATAAAAATAGGCGTATCACGAGGCAGAATTTCAGATAAAAAAAATCCTTAGCTTTCGCTAAGGATGATTTCTG

**B)** pAgBispA

## EcoRI

GAATTC

## Ptrc2O

CGAATTGTGAGCGCTCACAATTCGAACGGTTCTGGCAAATATTCTGAAATGAGCTGTTGACAATTAATCATCCGGCTCGTATAATGTGTGGAATTGTGAGCGGATAACAATTTCACACA

## Spacer

TACTCGAG

## **RBS***

TAGTGGAGGT

## XbaI

TCTAGA

## AgB

**ATG**GCCGGTGTGAGTGCCGTTAGCAAAGTGAGTTCTCTGGTGTGCGATCTGTCTAGCACTTCCGGGCTCATTCGGCGTACAGCCAACCCTCATCCAAATGTGTGGGGGTACGATCTGGTGCACTCTTTGAAATCTCCCTATATCGATTCTTCCTATCGTGAACGGGCTGAAGTGCTCGTTTCTGAGATCAAAGCCATGCTGAATCCCGCTATTACCGGCGACGGGGAATCCATGATTACCCCATCCGCCTATGATACTGCCTGGGTTGCCCGCGTTCCCGCAATTGATGGTAGCGCCCGTCCCCAATTTCCCCAAACGGTTGACTGGATTCTGAAGAACCAGTTAAAAGACGGCTCTTGGGGAATCCAATCCCACTTTCTATTGTCCGATCGGTTATTAGCCACCTTGAGCTGCGTCTTAGTATTATTGAAGTGGAACGTTGGCGATCTCCAAGTGGAACAGGGCATTGAATTTATTAAAAGTAATCTCGAGCTAGTCAAGGACGAAACGGATCAGGATAGTTTGGTGACAGATTTTGAGATTATTTTCCCCAGTTTACTGCGTGAAGCGCAGTCCCTGCGGTTGGGACTGCCGTATGACTTACCTTATATCCATTTGTTACAAACAAAACGACAAGAACGATTAGCTAAGCTGTCTCGCGAGGAAATCTACGCTGTGCCTAGTCCTCTGTTATACTCCTTAGAAGGTATTCAGGATATCGTCGAATGGGAACGTATTATGGAAGTTCAGAGTCAAGACGGAAGTTTTTTGAGCAGCCCAGCCTCCACTGCTTGTGTGTTTATGCATACCGGTGATGCCAAATGTTTAGAATTTCTCAATTCTGTGATGATCAAATTTGGCAATTTTGTGCCATGTTTATATCCAGTAGACTTGTTGGAACGGCTGTTGATTGTTGATAATATCGTCCGGTTGGGGATTTACCGTCATTTTGAGAAGGAGATTAAAGAAGCGCTGGATTATGTTTACCGCCATTGGAATGAGCGCGGGATTGGTTGGGGTCGGCTAAATCCCATTGCGGACTTAGAAACTACCGCATTGGGCTTTCGCTTGCTACGTCTGCATCGTTATAATGTGTCCCCCGCGATTTTTGATAACTTTAAAGATGCCAATGGCAAGTTTATTTGCAGCACAGGGCAGTTTAATAAAGATGTGGCGTCCATGTTAAATCTCTACCGCGCCTCTCAATTAGCCTTTCCTGGTGAAAATATTTTGGACGAAGCCAAATCTTTTGCGACGAAATACCTCCGCGAGGCGCTCGAAAAGAGCGAAACCTCCTCCGCTTGGAATAACAAACAGAATCTGTCCCAAGAAATTAAATATGCCTTAAAGACCTCCTGGCACGCAAGCGTACCTCGGGTTGAAGCTAAGCGCTATTGCCAGGTGTACCGCCCTGATTACGCCCGCATTGCCAAGTGTGTTTACAAACTCCCCTACGTCAACAACGAGAAATTCTTGGAACTCGGGAAATTAGACTTCAATATTATTCAGAGTATTCATCAAGAAGAAATGAAGAATGTAACCAGTTGGTTTCGTGACAGTGGTCTACCCTTGTTTACCTTTGCCCGAGAACGTCCCCTAGAGTTTTACTTTCTGGTGGCAGCGGGTACCTACGAACCCCAATACGCCAAGTGCCGGTTTTTGTTTACCAAAGTGGCCTGTTTACAAACCGTCCTCGATGACATGTATGATACCTATGGCACATTAGATGAATTAAAATTGTTTACCGAAGCCGTCCGTCGGTGGGACCTGAGTTTTACCGAAAATCTGCCCGATTACATGAAGTTATGCTATCAAATTTATTATGATATTGTGCACGAAGTTGCCTGGGAAGCCGAAAAAGAACAAGGCCGCGAATTGGTGAGCTTTTTTCGTAAAGGGTGGGAGGACTATCTATTGGGCTACTATGAAGAAGCTGAGTGGTTAGCCGCCGAATATGTGCCCACCTTAGATGAATATATTAAGAATGGCATCACCAGCATTGGACAACGCATTTTGTTGTTAAGCGGCGTTTTGATTATGGACGGCCAATTACTATCCCAAGAGGCTCTCGAAAAAGTCGACTACCCCGGGCGGCGTGTTCTAACTGAACTCAATTCTTTAATTTCCCGGTTAGCCGACGATACCAAAACATACAAAGCTGAAAAAGCCCGTGGCGAATTGGCCAGCAGTATTGAATGTTATATGAAAGATCACCCCGAATGTACAGAAGAAGAAGCTCTGGATCACATCTATAGCATCTTAGAACCGGCCGTCAAAGAGCTAACCCGCGAATTTTTAAAGCCAGACGATGTGCCCTTTGCGTGCAAAAAAATGCTCTTCGAAGAAACCCGTGTTACTATGGTGATTTTCAAAGATGGTGATGGTTTTGGAGTGAGCAAGCTAGAAGTTAAAGATCATATCAAGGAGTGCTTGATTGAACCTTTGCCCCTCCGGTCCGGTAGCGGAAGT

## FLAG-tag

GACTATAAGGATGATGATGACAAGGGA**TAG**

## SacII

CCGCGG

## Spacer

TAACACTGTATAACATTAAGA

## RBS

AGGAGGTAAA

## NdeI

CAT**ATG**

## ispA

GACTTTCCGCAGCAACTCGAAGCCTGCGTTAAGCAGGCCAACCAGGCGCTGAGCCGTTTTATCGCCCCACTGCCCTTTCAGAACACTCCCGTGGTCGAAACCATGCAGTATGGCGCATTATTAGGTGGTAAGCGCCTGCGACCTTTCCTGGTTTATGCCACCGGTCATATGTTCGGCGTTAGCACAAACACGCTGGACGCACCCGCTGCCGCCGTTGAGTGTATCCACGCTTACTCATTAATTCATGATGATTTACCGGCAATGGATGATGACGATCTGCGTCGCGGTTTGCCAACCTGCCATGTGAAGTTTGGCGAAGCAAACGCGATTCTCGCTGGCGACGCTTTACAAACGCTGGCGTTCTCGATTTTAAGCGATGCCGATATGCCGGAAGTGTCGGACCGCGACAGAATTTCGATGATTTCTGAACTGGCGAGCGCCAGTGGTATTGCCGGAATGTGCGGTGGTCAGGCATTAGATTTAGACGCGGAAGGCAAACACGTACCTCTGGACGCGCTTGAGCGTATTCATCGTCATAAAACCGGCGCATTGATTCGCGCCGCCGTTCGCCTTGGTGCATTAAGCGCCGGAGATAAAGGACGTCGTGCTCTGCCGGTACTCGACAAGTATGCAGAGAGCATCGGCCTTGCCTTCCAGGTTCAGGATGACATCCTGGATGTGGTGGGAGATACTGCAACGTTGGGAAAACGCCAGGGTGCCGACCAGCAACTTGGTAAAAGTACCTACCCTGCACTTCTGGGTCTTGAGCAAGCCCGGAAGAAAGCCCGGGATCTGATCGACGATGCCCGTCAGTCGCTGAAACAACTGGCTGAACAGTCACTCGATACCTCGGCACTGGAAGCGCTAGCGGACTACATCATCCAGCGTAATAAA

## BglII

AGATCT

## Gly-Ser linker

GGTAGCGGAAGT

## Strep-tag

TGGAGTCATCCTCAGTTCGAGAAG**TAA**

## SpeI

ACTAGT

TA

## SacII

CCGCGG

CCAGGCAT

## rrnB T1 Terminator

CAAATAAAACGAAAGGCTCAGTCGAAAGACTGGGCCTTTCGTTTTATCTGTTGTTTGTCGGTGAACGCTCTC

TACTAGAGTCACACT

## T7Te Terminator

GGCTCACCTTCGGGTGGGCCTTTCTGCG

TTTATA

## PstI

CTGCAG

## pPMQC1

TCCGGCAAAAAAGGGCAAGGTGTCACCACCCTGCCCTTTTTCTTTAAAACCGAAAAGATTACTTCGCGTTATGCAGGCTTCCTCGCTCACTGACTCGCTGCGCTCGGTCGTTCGGCTGCGGCGAGCGGTATCAGCTCACTCAAAGGCGGTAATCAATTGAGTTCTTTTACCCTCAGCCGAAATGCCTGCCGTTGCTAGACATTGCCAGCCAGTGCCCGTCACTCCCGTACTAACTGTCACGAACCCCTGCAATAACTGTCACGCCCCCCTGCAATAACTGTCACGAACCCCTGCAATAACTGTCACGCCCCCAAACCTGCAAACCCAGCAGGGGCGGGGGCTGGCGGGGTGTTGGAAAAATCCATCCATGATTATCTAAGAATAATCCACTAGGCGCGGTTATCAGCGCCCTTGTGGGGCGCTGCTGCCCTTGCCCAATATGCCCGGCCAGAGGCCGGATAGCTGGTCTATTCGCTGCGCTAGGCTACACACCGCCCCACCGCTGCGCGGCAGGGGGAAAGGCGGGCAAAGCCCGCTAAACCCCACACCAAACCCCGCAGAAATACGCTGGAGCGCTTTTAGCCGCTTTAGCGGCCTTTCCCCCTACCCGAAGGGTGGGGGCGCGTGTGCAGCCCCGCAGGGCCTGTCTCGGTCGATCATTCAGCCCGGCTCATCCTTCTGGCGTGGCGGCAGACCGAACAAGGCGCGGTCGTGGTCGCGTTCAAGGTACGCATCCATTGCCGCCATGAGCCGATCCTCCGGCCACTCGCTGCTGTTCACCTTGGCCAAAATCATGGCCCCCACCAGCACCTTGCGCCTTGTTTCGTTCTTGCGCTCTTGCTGCTGTTCCCTTGCCCGCACCCGCTGAATTTCGGCATTGATTCGCGCTCGTTGTTCTTCGAGCTTGGCCAGCCGATCCGCCGCCTTGTTGCTCCCCTTAACCATCTTGACACCCCATTGTTAATGTGCTGTCTCGTAGGCTATCATGGAGGCACAGCGGCGGCAATCCCGACCCTACTTTGTAGGGGAGGGCGCACTTACCGGTTTCTCTTCGAGAAACTGGCCTAACGGCCACCCTTCGGGCGGTGCGCTCTCCGAGGGCCATTGCATGGAGCCGAAAAGCAAAAGCAACAGCGAGGCAGCATGGCGATTTATCACCTTACGGCGAAAACCGGCAGCAGGTCGGGCGGCCAATCGGCCAGGGCCAAGGCCGACTACATCCAGCGCGAAGGCAAGTATGCCCGCGACATGGATGAAGTCTTGCACGCCGAATCCGGGCACATGCCGGAGTTCGTCGAGCGGCCCGCCGACTACTGGGATGCTGCCGACCTGTATGAACGCGCCAATGGGCGGCTGTTCAAGGAGGTCGAATTTGCCCTGCCGGTCGAGCTGACCCTCGACCAGCAGAAGGCGCTGGCGTCCGAGTTCGCCCAGCACCTGACCGGTGCCGAGCGCCTGCCGTATACGCTGGCCATCCATGCCGGTGGCGGCGAGAACCCGCACTGCCACCTGATGATCTCCGAGCGGATCAATGACGGCATCGAGCGGCCCGCCGCTCAGTGGTTCAAGCGGTACAACGGCAAGACCCCGGAGAAGGGCGGGGCACAGAAGACCGAAGCGCTCAAGCCCAAGGCATGGCTTGAGCAGACCCGCGAGGCATGGGCCGACCATGCCAACCGGGCATTAGAGCGGGCTGGCCACGACGCCCGCATTGACCACAGAACACTTGAGGCGCAGGGCATCGAGCGCCTGCCCGGTGTTCACCTGGGGCCGAACGTGGTGGAGATGGAAGGCCGGGGCATCCGCACCGACCGGGCAGACGTGGCCCTGAACATCGACACCGCCAACGCCCAGATCATCGACTTACAGGAATACCGGGAGGCAATAGACCATGAACGCAATCGACAGAGTGAAGAAATCCAGAGGCATCAACGAGTTAGCGGAGCAGATCGAACCGCTGGCCCAGAGCATGGCGACACTGGCCGACGAAGCCCGGCAGGTCATGAGCCAGACCCAGCAGGCCAGCGAGGCGCAGGCGGCGGAGTGGCTGAAAGCCCAGCGCCAGACAGGGGCGGCATGGGTGGAGCTGGCCAAAGAGTTGCGGGAGGTAGCCGCCGAGGTGAGCAGCGCCGCGCAGAGCGCCCGGAGCGCGTCGCGGGGGTGGCACTGGAAGCTATGGCTAACCGTGATGCTGGCTTCCATGATGCCTACGGTGGTGCTGCTGATCGCATCGTTGCTCTTGCTCGACCTGACGCCACTGACAACCGAGGACGGCTCGATCTGGCTGCGCTTGGTGGCCCGATGAAGAACGACAGGACTTTGCAGGCCATAGGCCGACAGCTCAAGGCCATGGGCTGTGAGCGCTTCGATATCGGCGTCAGGGACGCCACCACCGGCCAGATGATGAACCGGGAATGGTCAGCCGCCGAAGTGCTCCAGAACACGCCATGGCTCAAGCGGATGAATGCCCAGGGCAATGACGTGTATATCAGGCCCGCCGAGCAGGAGCGGCATGGTCTGGTGCTGGTGGACGACCTCAGCGAGTTTGACCTGGATGACATGAAAGCCGAGGGCCGGGAGCCTGCCCTGGTAGTGGAAACCAGCCCGAAGAACTATCAGGCATGGGTCAAGGTGGCCGACGCCGCAGGCGGTGAACTTCGGGGGCAGATTGCCCGGACGCTGGCCAGCGAGTACGACGCCGACCCGGCCAGCGCCGACAGCCGCCACTATGGCCGCTTGGCGGGCTTCACCAACCGCAAGGACAAGCACACCACCCGCGCCGGTTATCAGCCGTGGGTGCTGCTGCGTGAATCCAAGGGCAAGACCGCCACCGCTGGCCCGGCGCTGGTGCAGCAGGCTGGCCAGCAGATCGAGCAGGCCCAGCGGCAGCAGGAGAAGGCCCGCAGGCTGGCCAGCCTCGAACTGCCCGAGCGGCAGCTTAGCCGCCACCGGCGCACGGCGCTGGACGAGTACCGCAGCGAGATGGCCGGGCTGGTCAAGCGCTTCGGTGATGACCTCAGCAAGTGCGACTTTATCGCCGCGCAGAAGCTGGCCAGCCGGGGCCGCAGTGCCGAGGAAATCGGCAAGGCCATGGCCGAGGCCAGCCCAGCGCTGGCAGAGCGCAAGCCCGGCCACGAAGCGGATTACATCGAGCGCACCGTCAGCAAGGTCATGGGTCTGCCCAGCGTCCAGCTTGCGCGGGCCGAGCTGGCACGGGCACCGGCACCCCGCCAGCGAGGCATGGACAGGGGCGGGCCAGATTTCAGCATGTAGTGCTTGCGTTGGTACTCACGCCTGTTATACTATGAGTACTCACGCACAGAAGGGGGTTTTATGGAATACGAAAAAAGCGCTTCAGGGTCGGTCTACCTGATCAAAAGTGACAAGGGCTATTGGTTGCCCGGTGGCTTTGGTTATACGTCAAACAAGGCCGAGGCTGGCCGCTTTTCAGTCGCTGATATGGCCAGCCTTAACCTTGACGGCTGCACCTTGTCCTTGTTCCGCGAAGACAAGCCTTTCGGCCCCGGCAAGTTTCTCGGTGACTGATATGAAAGACCAAAAGGACAAGCAGACCGGCGACCTGCTGGCCAGCCCTGACGCTGTACGCCAAGCGCGATATGCCGAGCGCATGAAGGCCAAAGGGATGCGTCAGCGCAAGTTCTGGCTGACCGACGACGAATACGAGGCGCTGCGCGAGTGCCTGGAAGAACTCAGAGCGGCGCAGGGCGGGGGTAGTGACCCCGCCAGCGCCTAACCACCAACTGCCTGCAAAGGAGGCAATCAATGGCTACCCATAAGCCTATCAATATTCTGGAGGCGTTCGCAGCAGCGCCGCCACCGCTGGACTACGTTTTGCCCAACATGGTGGCCGGTACGGTCGGGGCGCTGGTGTCGCCCGGTGGTGCCGGTAAATCCATGCTGGCCCTGCAACTGGCCGCACAGATTGCAGGCGGGCCGGATCTGCTGGAGGTGGGCGAACTGCCCACCGGCCCGGTGATCTACCTGCCCGCCGAAGACCCGCCCACCGCCATTCATCACCGCCTGCACGCCCTTGGGGCGCACCTCAGCGCCGAGGAACGGCAAGCCGTGGCTGACGGCCTGCTGATCCAGCCGCTGATCGGCAGCCTGCCCAACATCATGGCCCCGGAGTGGTTCGACGGCCTCAAGCGCGCCGCCGAGGGCCGCCGCCTGATGGTGCTGGACACGCTGCGCCGGTTCCACATCGAGGAAGAAAACGCCAGCGGCCCCATGGCCCAGGTCATCGGTCGCATGGAGGCCATCGCCGCCGATACCGGGTGCTCTATCGTGTTCCTGCACCATGCCAGCAAGGGCGCGGCCATGATGGGCGCAGGCGACCAGCAGCAGGCCAGCCGGGGCAGCTCGGTACTGGTCGATAACATCCGCTGGCAGTCCTACCTGTCGAGCATGACCAGCGCCGAGGCCGAGGAATGGGGTGTGGACGACGACCAGCGCCGGTTCTTCGTCCGCTTCGGTGTGAGCAAGGCCAACTATGGCGCACCGTTCGCTGATCGGTGGTTCAGGCGGCATGACGGCGGGGTGCTCAAGCCCGCCGTGCTGGAGAGGCAGCGCAAGAGCAAGGGGGTGCCCCGTGGTGAAGCCTAAGAACAAGCACAGCCTCAGCCACGTCCGGCACGACCCGGCGCACTGTCTGGCCCCCGGCCTGTTCCGTGCCCTCAAGCGGGGCGAGCGCAAGCGCAGCAAGCTGGACGTGACGTATGACTACGGCGACGGCAAGCGGATCGAGTTCAGCGGCCCGGAGCCGCTGGGCGCTGATGATCTGCGCATCCTGCAAGGGCTGGTGGCCATGGCTGGGCCTAATGGCCTAGTGCTTGGCCCGGAACCCAAGACCGAAGGCGGACGGCAGCTCCGGCTGTTCCTGGAACCCAAGTGGGAGGCCGTCACCGCTGATGCCATGGTGGTCAAAGGTAGCTATCGGGCGCTGGCAAAGGAAATCGGGGCAGAGGTCGATAGTGGTGGGGCGCTCAAGCACATACAGGACTGCATCGAGCGCCTTTGGAAGGTATCCATCATCGCCCAGAATGGCCGCAAGCGGCAGGGGTTTCGGCTGCTGTCGGAGTACGCCAGCGACGAGGCGGACGGGCGCCTGTACGTGGCCCTGAACCCCTTGATCGCGCAGGCCGTCATGGGTGGCGGCCAGCATGTGCGCATCAGCATGGACGAGGTGCGGGCGCTGGACAGCGAAACCGCCCGCCTGCTGCACCAGCGGCTGTGTGGCTGGATCGACCCCGGCAAAACCGGCAAGGCTTCCATAGATACCTTGTGCGGCTATGTCTGGCCGTCAGAGGCCAGTGGTTCGACCATGCGCAAGCGCCGCCAGCGGGTGCGCGAGGCGTTGCCGGAGCTGGTCGCGCTGGGCTGGACGGTAACCGAGTTCGCGGCGGGCAAGTACGACATCACCCGGCCCAAGGCGGCAGGCTGACCCCCCCCACTCTATTGTAAACAAGACATTTTTATCTTTTATATTCAATGGCTTATTTTCCTGCTAATCAGCTCGAGGCTTGGATTCTCACCAATAAAAAACGCCCGGCGGCAACCGAGCGTTCTGAACAAATCCAGATGGAGTTCTGAGGTCATTACTGGATCTATCAACAGGAGTCCAAGCGAGCTCGATATCAAATTACGCCCCGCCCTGCCACTCATCGCAGTACTGTTGTAATTCATTAAGCATTCTGCCGACATGGAAGCCATCACAAACGGCATGATGAACCTGAATCGCCAGCGGCATCAGCACCTTGTCGCCTTGCGTATAATATTTGCCCATGGTGAAAACGGGGGCGAAGAAGTTGTCCATATTGGCCACGTTTAAATCAAAACTGGTGAAACTCACCCAGGGATTGGCTGAGACGAAAAACATATTCTCAATAAACCCTTTAGGGAAATAGGCCAGGTTTTCACCGTAACACGCCACATCTTGCGAATATATGTGTAGAAACTGCCGGAAATCGTCGTGGTATTCACTCCAGAGCGATGAAAACGTTTCAGTTTGCTCATGGAAAACGGTGTAACAAGGGTGAACACTATCCCATATCACCAGCTCACCGTCTTTCATTGCCATACGAAATTCCGGATGAGCATTCATCAGGCGGGCAAGAATGTGAATAAAGGCCGGATAAAACTTGTGCTTATTTTTCTTTACGGTCTTTAAAAAGGCCGTAATATCCAGCTGAACGGTCTGGTTATAGGTACATTGAGCAACTGACTGAAATGCCTCAAAATGTTCTTTACGATGCCATTGGGATATATCAACGGTGGTATATCCAGTGATTTTTTTCTCCATTTTAGCTTCCTTAGCTCCTGAAAATCTCGATAACTCAAAAAATACGCCCGGTAGTGATCTTATTTCATTATGGTGAAAGTTGGAACCTCTTACGTGCCCGATCAACTCGAGTGCCACCTGACGTCTAAGAAACCATTATTATCATGACATTAACCTATAAAAATAGGCGTATCACGAGGCAGAATTTCAGATAAAAAAAATCCTTAGCTTTCGCTAAGGATGATTTCTG

**C)** p2MEP-AgBispA

## EcoRI

GAATTC

## Ptrc core

GAGCTGTTGACAATTGTGAGCGCTCACAATATAATGTGTGGAA

## BCD2

GGGCCCAAGTTCACTTAAAAAGGAGATCAACA**ATG**AAAGCAATTTTCGTACTGAAACATCTTAATCATGCTAAGGAGGTTTTC**TAATG**

## Strep-tag

TGGAGTCATCCTCAGTTCGAGAAG

## Gly-Ser linker

GGTAGCGGAAGT

## Scar

GGATCT

## sIdi

**ATG**GACTCCACTCCCCACCGCAAGTCTGACCATATCCGGATTGTGTTAGAGGAAGATGTCGTGGGCAAGGGAATTTCCACTGGTTTTGAGCGACTCATGCTTGAACACTGTGCCCTACCCGCCGTCGACCTGGATGCAGTTGACCTGGGCTTGACCTTGTGGGGGAAAAGTTTAACTTATCCATGGCTAATTTCGAGTATGACAGGCGGTACTCCAGAAGCTAAGCAAATCAATCTATTTCTTGCTGAAGTCGCGCAAGCTCTCGGCATTGCCATGGGCCTGGGCAGCCAAAGGGCCGCTATCGAAAATCCCGATCTAGCGTTTACCTACCAAGTGAGGTCAGTGGCGCCTGACATTTTGCTGTTTGCGAACTTGGGCTTAGTCCAATTGAATTATGGTTATGGGTTGGAACAGGCCCAACGAGCCGTGGACATGATTGAAGCTGATGCTCTGATTCTACATTTGAATCCACTTCAAGAAGCCGTGCAACCTGACGGTGACCGACTTTGGTCTGGCTTATGGTCCAAATTGGAGGCCCTGGTCGAAGCTCTGGAAGTGCCCGTCATTGTGAAAGAAGTCGGGAACGGTATCTCTGGTCCCGTGGCTAAACGCCTACAAGAATGTGGCGTAGGAGCCATTGACGTAGCCGGAGCTGGCGGTACCAGTTGGTCGGAAGTGGAAGCTCATCGGCAGACCGATCGCCAGGCCAAAGAAGTGGCGCATAACTTTGCGGATTGGGGGCTGCCCACTGCCTGGTCCCTTCAGCAGGTGGTGCAGAATACCGAACAAATTCTCGTGTTCGCGAGCGGGGGTATTCGGTCTGGTATTGATGGGGCGAAAGCCATAGCGCTTGGTGCCACCCTGGTTGGTTCTGCCGCACCTGTTCTTGCCGAAGCCAAGATTAATGCTCAAAGGGTGTACGACCACTACCAAGCCCGGTTACGCGAATTGCAGATTGCGGCTTTCTGTTGTGATGCCGCAAATCTTACCCAATTGGCGCAGGTTCCTTTATGGGACAGGCAAAGCGGGCAGCGACTCACCAAGCCC**TAA**

## Scar

TCTAGT

## RBS

AAAGAGGAGAAA

CTAGA

## Strep-tag

**ATG**TGGAGCCATCCTCAATTTGAAAAG

## Gly-Ser linker

GGCAGTGGTTCAGGTAGT

## CfDXS

**ATG**GCTGCTCTGTACCAGGATAACACGAACGATGTCGTTCCGAGTGGAGAGGGTCTGACGAGGCAGAAACCAAGAACTCTGAGTTTCACGGGAGAGAAGCCTTCAACTCCAATTTTGGATACCATCAACTATCCAATCCACATGAAGAATCTGTCCGTGGAGGAACTGGAGATATTGGCCGATGAACTGAGGGAGGAGATAGTTTACACGGTGTCGAAAACGGGAGGGCATTTGAGCTCAAGCTTGGGTGTATCAGAGCTCACCGTTGCACTGCATCATGTATTCAACACACCCGATGACAAAATCATCTGGGATGTTGGACATCAGGCGTATCCACACAAAATCTTGACAGGGAGGAGGTCCAGAATGCACACCATCCGACAGACTTTCGGGCTTGCAGGGTTCCCCAAGAGGGATGAGAGCCCGCACGACGCGTTCGGAGCTGGTCACAGCTCCACCAGTATTTCAGCTGGTCTAGGGATGGCGGTGGGGAGGGACTTGCTACAGAAGAACAACCACGTGATCTCGGTGATCGGAGACGGAGCCATGACAGCGGGGCAGGCATACGAGGCCATGAACAATGCAGGATTTCTTGATTCCAATCTGATCATCGTGTTGAACGACAACAAACAAGTGTCCCTGCCTACAGCCACCGTCGACGGCCCTGCTCCTCCCGTCGGAGCCTTGAGCAAAGCCCTCACCAAGCTGCAAGCAAGCAGGAAGTTCCGGCAGCTACGAGAAGCAGCAAAAGGCATGACTAAGCAGATGGGAAACCAAGCACACGAAATTGCATCCAAGGTAGACACTTACGTTAAAGGAATGATGGGGAAACCAGGCGCCTCCCTCTTCGAGGAGCTCGGGATTTATTACATCGGCCCTGTAGATGGACATAACATCGAAGATCTTGTCTATATTTTCAAGAAAGTTAAGGAGATGCCTGCGCCCGGCCCTGTTCTTATTCACATCATCACCGAGAAGGGCAAAGGCTACCCTCCAGCTGAAGTTGCTGCTGACAAAATGCATGGTGTGGTGAAGTTTGATCCAACAACGGGGAAACAGATGAAGGTGAAAACGAAGACTCAATCATACACCCAATACTTCGCGGAGTCTCTGGTTGCAGAAGCAGAGCAGGACGAGAAAGTGGTGGCGATCCACGCGGCGATGGGAGGCGGAACGGGGCTGAACATCTTCCAGAAACGGTTTCCCGACCGATGTTTCGATGTCGGGATAGCCGAGCAGCATGCAGTCACCTTCGCTGCGGGTCTTGCAACGGAAGGCCTCAAGCCCTTCTGCACAATCTACTCTTCCTTCCTACAGCGAGGTTATGATCAGGTGGTGCACGATGTGGATCTTCAGAAACTCCCGGTGAGATTCATGATGGACAGAGCTGGACTTGTGGGAGCTGACGGCCCAACCCATTGCGGCGCCTTCGACACCACCTACATGGCCTGCCTGCCCAACATGGTCGTCATGGCTCCCTCCGATGAGGCTGAGCTCATGCACATGGTCGCCACTGCCGCTGTCATTGATGATCGCCCTAGCTGCGTTAGGTACCCTAGAGGAAACGGTATAGGGGTGCCCCTCCCTCCAAACAATAAAGGAATTCCATTAGAGGTTGGGAAGGGAAGGATTTTGAAAGAGGGTAACCGAGTTGCCATTCTAGGCTTCGGAACTATCGTGCAAAACTGTCTAGCAGCAGCCCAACTTCTTCAAGAACACGGCATATCCGTGAGCGTAGCCGATGCGAGATTCTGCAAGCCTCTGGATGGAGATCTGATCAAGAATCTTGTGAAGGAGCACGAAGTTCTCATCACTGTGGAAGAGGGTTCCATTGGAGGATTCAGTGCACATGTCTCTCATTTCTTGTCCCTCAATGGACTCCTCGACGGCAATCTTAAGTGGAGGCCTATGGTGCTCCCAGATAGGTACATTGATCATGGAGCATACCCTGATCAGATTGAGGAAGCAGGGCTGAGCTCAAAGCATATTGCAGGAACTGTTTTGTCACTTATTGGTGGAGGGAAAGACAGTCTTCATTTGATCAACATG**TAA**

## Scar

TCTAGT

## Terminator

## CCAGGCATCAAATAAAACGAAAGGCTCAGTCGAAAGACTGGGCCTTTCGTTTTATCTGTTGTTTGTCGGTGAACGCTCTCTACTAGAGTCACACTGGCTCACCTTCGGGTGGGCCTTTCTGCGTTTATA

## Scar

TCTAGT

AAGCA

## Scar

TCTAGT

**TTA**

## Strep-Tag

CTTCTCGAACTGAGGATGACTCCA

## Gly-Ser linker

ACTTCCGCTACC

## BglII

AGATCT

## ispA

TTTATTACGCTGGATGATGTAGTCCGCTAGCGCTTCCAGTGCCGAGGTATCGAGTGACTGTTCAGCCAGTTGTTTCAGCGACTGACGGGCATCGTCGATCAGATCCCGGGCTTTCTTCCGGGCTTGCTCAAGACCCAGAAGTGCAGGGTAGGTACTTTTACCAAGTTGCTGGTCGGCACCCTGGCGTTTTCCCAACGTTGCAGTATCTCCCACCACATCCAGGATGTCATCCTGAACCTGGAAGGCAAGGCCGATGCTCTCTGCATACTTGTCGAGTACCGGCAGAGCACGACGTCCTTTATCTCCGGCGCTTAATGCACCAAGGCGAACGGCGGCGCGAATCAATGCGCCGGTTTTATGACGATGAATACGCTCAAGCGCGTCCAGAGGTACGTGTTTGCCTTCCGCGTCTAAATCTAATGCCTGACCACCGCACATTCCGGCAATACCACTGGCGCTCGCCAGTTCAGAAATCATCGAAATTCTGTCGCGGTCCGACACTTCCGGCATATCGGCATCGCTTAAAATCGAGAACGCCAGCGTTTGTAAAGCGTCGCCAGCGAGAATCGCGTTTGCTTCGCCAAACTTCACATGGCAGGTTGGCAAACCGCGACGCAGATCGTCATCATCCATTGCCGGTAAATCATCATGAATTAATGAGTAAGCGTGGATACACTCAACGGCGGCAGCGGGTGCGTCCAGCGTGTTTGTGCTAACGCCGAACATATGACCGGTGGCATAAACCAGGAAAGGTCGCAGGCGCTTACCACCTAATAATGCGCCATACTGCATGGTTTCGACCACGGGAGTGTTCTGAAAGGGCAGTGGGGCGATAAAACGGCTCAGCGCCTGGTTGGCCTGCTTAACGCAGGCTTCGAGTTGCTGCGGAAAGTC**CAT**ATG

## RBS

TTTACCTCCT

## Spacer

TCTTAATGTTATACAGTGTTA

## SacII

CCGCGG

**CTA**

## FLAG

TCCCTTGTCATCATCATCCTTATAGTC

## Gly-Ser Linker

ACTTCCGCTACCGGA

## AgB

CCGGAGGGGCAAAGGTTCAATCAAGCACTCCTTGATATGATCTTTAACTTCTAGCTTGCTCACTCCAAAACCATCACCATCTTTGAAAATCACCATAGTAACACGGGTTTCTTCGAAGAGCATTTTTTTGCACGCAAAGGGCACATCGTCTGGCTTTAAAAATTCGCGGGTTAGCTCTTTGACGGCCGGTTCTAAGATGCTATAGATGTGATCCAGAGCTTCTTCTTCTGTACATTCGGGGTGATCTTTCATATAACATTCAATACTGCTGGCCAATTCGCCACGGGCTTTTTCAGCTTTGTATGTTTTGGTATCGTCGGCTAACCGGGAAATTAAAGAATTGAGTTCAGTTAGAACACGCCGCCCGGGGTAGTCGACTTTTTCGAGAGCCTCTTGGGATAGTAATTGGCCGTCCATAATCAAAACGCCGCTTAACAACAAAATGCGTTGTCCAATGCTGGTGATGCCATTCTTAATATATTCATCTAAGGTGGGCACATATTCGGCGGCTAACCACTCAGCTTCTTCATAGTAGCCCAATAGATAGTCCTCCCACCCTTTACGAAAAAAGCTCACCAATTCGCGGCCTTGTTCTTTTTCGGCTTCCCAGGCAACTTCGTGCACAATATCATAATAAATTTGATAGCATAACTTCATGTAATCGGGCAGATTTTCGGTAAAACTCAGGTCCCACCGACGGACGGCTTCGGTAAACAATTTTAATTCATCTAATGTGCCATAGGTATCATACATGTCATCGAGGACGGTTTGTAAACAGGCCACTTTGGTAAACAAAAACCGGCACTTGGCGTATTGGGGTTCGTAGGTACCCGCTGCCACCAGAAAGTAAAACTCTAGGGGACGTTCTCGGGCAAAGGTAAACAAGGGTAGACCACTGTCACGAAACCAACTGGTTACATTCTTCATTTCTTCTTGATGAATACTCTGAATAATATTGAAGTCTAATTTCCCGAGTTCCAAGAATTTCTCGTTGTTGACGTAGGGGAGTTTGTAAACACACTTGGCAATGCGGGCGTAATCAGGGCGGTACACCTGGCAATAGCGCTTAGCTTCAACCCGAGGTACGCTTGCGTGCCAGGAGGTCTTTAAGGCATATTTAATTTCTTGGGACAGATTCTGTTTGTTATTCCAAGCGGAGGAGGTTTCGCTCTTTTCGAGCGCCTCGCGGAGGTATTTCGTCGCAAAAGATTTGGCTTCGTCCAAAATATTTTCACCAGGAAAGGCTAATTGAGAGGCGCGGTAGAGATTTAACATGGACGCCACATCTTTATTAAACTGCCCTGTGCTGCAAATAAACTTGCCATTGGCATCTTTAAAGTTATCAAAAATCGCGGGGGACACATTATAACGATGCAGACGTAGCAAGCGAAAGCCCAATGCGGTAGTTTCTAAGTCCGCAATGGGATTTAGCCGACCCCAACCAATCCCGCGCTCATTCCAATGGCGGTAAACATAATCCAGCGCTTCTTTAATCTCCTTCTCAAAATGACGGTAAATCCCCAACCGGACGATATTATCAACAATCAACAGCCGTTCCAACAAGTCTACTGGATATAAACATGGCACAAAATTGCCAAATTTGATCATCACAGAATTGAGAAATTCTAAACATTTGGCATCACCGGTATGCATAAACACACAAGCAGTGGAGGCTGGGCTGCTCAAAAAACTTCCGTCTTGACTCTGAACTTCCATAATACGTTCCCATTCGACGATATCCTGAATACCTTCTAAGGAGTATAACAGAGGACTAGGCACAGCGTAGATTTCCTCGCGAGACAGCTTAGCTAATCGTTCTTGTCGTTTTGTTTGTAACAAATGGATATAAGGTAAGTCATACGGCAGTCCCAACCGCAGGGACTGCGCTTCACGCAGTAAACTGGGGAAAATAATCTCAAAATCTGTCACCAAACTATCCTGATCCGTTTCGTCCTTGACTAGCTCGAGATTACTTTTAATAAATTCAATGCCCTGTTCCACTTGGAGATCGCCAACGTTCCACTTCAATAATACTAAGACGCAGCTCAAGGTGGCTAATAACCGATCGGACAATAGAAAGTGGGATTGGATTCCCCAAGAGCCGTCTTTTAACTGGTTCTTCAGAATCCAGTCAACCGTTTGGGGAAATTGGGGACGGGCGCTACCATCAATTGCGGGAACGCGGGCAACCCAGGCAGTATCATAGGCGGATGGGGTAATCATGGATTCCCCGTCGCCGGTAATAGCGGGATTCAGCATGGCTTTGATCTCAGAAACGAGCACTTCAGCCCGTTCACGATAGGAAGAATCGATATAGGGAGATTTCAAAGAGTGCACCAGATCGTACCCCCACACATTTGGATGAGGGTTGGCTGTACGCCGAATGAGCCCGGAAGTGCTAGACAGATCGCACACCAGAGAACTCACTTTGCTAACGGCACTCACACCGGC**CAT**

## XbaI

TCTAGA

## RBS

ACCTCCACTA

## Spacer

CTCGAGTA

## Ptrc2O

TGTGTGAAATTGTTATCCGCTCACAATTCCACACATTATACGAGCCGGATGATTAATTGTCAACAGCTCATTTCAGAATATTTGCCAGAACCGTTCGAATTGTGAGCGCTCACAATTCG

## EcoRI

GAATTC

CAGAAATCATCCTTAGCGAAAGCTAAGGATTTTTTTTATCTGAAATTCTGCCTCGTGATACGCCTATTTTTATAGGTTAATGTCATGATAATAATGGTTTCTTAGACGTCAGGTGGCACTCGAGTTGATCGGGCACGTAAG

## PstI

CTGCAG

## pPMQC1

TCCGGCAAAAAAGGGCAAGGTGTCACCACCCTGCCCTTTTTCTTTAAAACCGAAAAGATTACTTCGCGTTATGCAGGCTTCCTCGCTCACTGACTCGCTGCGCTCGGTCGTTCGGCTGCGGCGAGCGGTATCAGCTCACTCAAAGGCGGTAATCAATTGAGTTCTTTTACCCTCAGCCGAAATGCCTGCCGTTGCTAGACATTGCCAGCCAGTGCCCGTCACTCCCGTACTAACTGTCACGAACCCCTGCAATAACTGTCACGCCCCCCTGCAATAACTGTCACGAACCCCTGCAATAACTGTCACGCCCCCAAACCTGCAAACCCAGCAGGGGCGGGGGCTGGCGGGGTGTTGGAAAAATCCATCCATGATTATCTAAGAATAATCCACTAGGCGCGGTTATCAGCGCCCTTGTGGGGCGCTGCTGCCCTTGCCCAATATGCCCGGCCAGAGGCCGGATAGCTGGTCTATTCGCTGCGCTAGGCTACACACCGCCCCACCGCTGCGCGGCAGGGGGAAAGGCGGGCAAAGCCCGCTAAACCCCACACCAAACCCCGCAGAAATACGCTGGAGCGCTTTTAGCCGCTTTAGCGGCCTTTCCCCCTACCCGAAGGGTGGGGGCGCGTGTGCAGCCCCGCAGGGCCTGTCTCGGTCGATCATTCAGCCCGGCTCATCCTTCTGGCGTGGCGGCAGACCGAACAAGGCGCGGTCGTGGTCGCGTTCAAGGTACGCATCCATTGCCGCCATGAGCCGATCCTCCGGCCACTCGCTGCTGTTCACCTTGGCCAAAATCATGGCCCCCACCAGCACCTTGCGCCTTGTTTCGTTCTTGCGCTCTTGCTGCTGTTCCCTTGCCCGCACCCGCTGAATTTCGGCATTGATTCGCGCTCGTTGTTCTTCGAGCTTGGCCAGCCGATCCGCCGCCTTGTTGCTCCCCTTAACCATCTTGACACCCCATTGTTAATGTGCTGTCTCGTAGGCTATCATGGAGGCACAGCGGCGGCAATCCCGACCCTACTTTGTAGGGGAGGGCGCACTTACCGGTTTCTCTTCGAGAAACTGGCCTAACGGCCACCCTTCGGGCGGTGCGCTCTCCGAGGGCCATTGCATGGAGCCGAAAAGCAAAAGCAACAGCGAGGCAGCATGGCGATTTATCACCTTACGGCGAAAACCGGCAGCAGGTCGGGCGGCCAATCGGCCAGGGCCAAGGCCGACTACATCCAGCGCGAAGGCAAGTATGCCCGCGACATGGATGAAGTCTTGCACGCCGAATCCGGGCACATGCCGGAGTTCGTCGAGCGGCCCGCCGACTACTGGGATGCTGCCGACCTGTATGAACGCGCCAATGGGCGGCTGTTCAAGGAGGTCGAATTTGCCCTGCCGGTCGAGCTGACCCTCGACCAGCAGAAGGCGCTGGCGTCCGAGTTCGCCCAGCACCTGACCGGTGCCGAGCGCCTGCCGTATACGCTGGCCATCCATGCCGGTGGCGGCGAGAACCCGCACTGCCACCTGATGATCTCCGAGCGGATCAATGACGGCATCGAGCGGCCCGCCGCTCAGTGGTTCAAGCGGTACAACGGCAAGACCCCGGAGAAGGGCGGGGCACAGAAGACCGAAGCGCTCAAGCCCAAGGCATGGCTTGAGCAGACCCGCGAGGCATGGGCCGACCATGCCAACCGGGCATTAGAGCGGGCTGGCCACGACGCCCGCATTGACCACAGAACACTTGAGGCGCAGGGCATCGAGCGCCTGCCCGGTGTTCACCTGGGGCCGAACGTGGTGGAGATGGAAGGCCGGGGCATCCGCACCGACCGGGCAGACGTGGCCCTGAACATCGACACCGCCAACGCCCAGATCATCGACTTACAGGAATACCGGGAGGCAATAGACCATGAACGCAATCGACAGAGTGAAGAAATCCAGAGGCATCAACGAGTTAGCGGAGCAGATCGAACCGCTGGCCCAGAGCATGGCGACACTGGCCGACGAAGCCCGGCAGGTCATGAGCCAGACCCAGCAGGCCAGCGAGGCGCAGGCGGCGGAGTGGCTGAAAGCCCAGCGCCAGACAGGGGCGGCATGGGTGGAGCTGGCCAAAGAGTTGCGGGAGGTAGCCGCCGAGGTGAGCAGCGCCGCGCAGAGCGCCCGGAGCGCGTCGCGGGGGTGGCACTGGAAGCTATGGCTAACCGTGATGCTGGCTTCCATGATGCCTACGGTGGTGCTGCTGATCGCATCGTTGCTCTTGCTCGACCTGACGCCACTGACAACCGAGGACGGCTCGATCTGGCTGCGCTTGGTGGCCCGATGAAGAACGACAGGACTTTGCAGGCCATAGGCCGACAGCTCAAGGCCATGGGCTGTGAGCGCTTCGATATCGGCGTCAGGGACGCCACCACCGGCCAGATGATGAACCGGGAATGGTCAGCCGCCGAAGTGCTCCAGAACACGCCATGGCTCAAGCGGATGAATGCCCAGGGCAATGACGTGTATATCAGGCCCGCCGAGCAGGAGCGGCATGGTCTGGTGCTGGTGGACGACCTCAGCGAGTTTGACCTGGATGACATGAAAGCCGAGGGCCGGGAGCCTGCCCTGGTAGTGGAAACCAGCCCGAAGAACTATCAGGCATGGGTCAAGGTGGCCGACGCCGCAGGCGGTGAACTTCGGGGGCAGATTGCCCGGACGCTGGCCAGCGAGTACGACGCCGACCCGGCCAGCGCCGACAGCCGCCACTATGGCCGCTTGGCGGGCTTCACCAACCGCAAGGACAAGCACACCACCCGCGCCGGTTATCAGCCGTGGGTGCTGCTGCGTGAATCCAAGGGCAAGACCGCCACCGCTGGCCCGGCGCTGGTGCAGCAGGCTGGCCAGCAGATCGAGCAGGCCCAGCGGCAGCAGGAGAAGGCCCGCAGGCTGGCCAGCCTCGAACTGCCCGAGCGGCAGCTTAGCCGCCACCGGCGCACGGCGCTGGACGAGTACCGCAGCGAGATGGCCGGGCTGGTCAAGCGCTTCGGTGATGACCTCAGCAAGTGCGACTTTATCGCCGCGCAGAAGCTGGCCAGCCGGGGCCGCAGTGCCGAGGAAATCGGCAAGGCCATGGCCGAGGCCAGCCCAGCGCTGGCAGAGCGCAAGCCCGGCCACGAAGCGGATTACATCGAGCGCACCGTCAGCAAGGTCATGGGTCTGCCCAGCGTCCAGCTTGCGCGGGCCGAGCTGGCACGGGCACCGGCACCCCGCCAGCGAGGCATGGACAGGGGCGGGCCAGATTTCAGCATGTAGTGCTTGCGTTGGTACTCACGCCTGTTATACTATGAGTACTCACGCACAGAAGGGGGTTTTATGGAATACGAAAAAAGCGCTTCAGGGTCGGTCTACCTGATCAAAAGTGACAAGGGCTATTGGTTGCCCGGTGGCTTTGGTTATACGTCAAACAAGGCCGAGGCTGGCCGCTTTTCAGTCGCTGATATGGCCAGCCTTAACCTTGACGGCTGCACCTTGTCCTTGTTCCGCGAAGACAAGCCTTTCGGCCCCGGCAAGTTTCTCGGTGACTGATATGAAAGACCAAAAGGACAAGCAGACCGGCGACCTGCTGGCCAGCCCTGACGCTGTACGCCAAGCGCGATATGCCGAGCGCATGAAGGCCAAAGGGATGCGTCAGCGCAAGTTCTGGCTGACCGACGACGAATACGAGGCGCTGCGCGAGTGCCTGGAAGAACTCAGAGCGGCGCAGGGCGGGGGTAGTGACCCCGCCAGCGCCTAACCACCAACTGCCTGCAAAGGAGGCAATCAATGGCTACCCATAAGCCTATCAATATTCTGGAGGCGTTCGCAGCAGCGCCGCCACCGCTGGACTACGTTTTGCCCAACATGGTGGCCGGTACGGTCGGGGCGCTGGTGTCGCCCGGTGGTGCCGGTAAATCCATGCTGGCCCTGCAACTGGCCGCACAGATTGCAGGCGGGCCGGATCTGCTGGAGGTGGGCGAACTGCCCACCGGCCCGGTGATCTACCTGCCCGCCGAAGACCCGCCCACCGCCATTCATCACCGCCTGCACGCCCTTGGGGCGCACCTCAGCGCCGAGGAACGGCAAGCCGTGGCTGACGGCCTGCTGATCCAGCCGCTGATCGGCAGCCTGCCCAACATCATGGCCCCGGAGTGGTTCGACGGCCTCAAGCGCGCCGCCGAGGGCCGCCGCCTGATGGTGCTGGACACGCTGCGCCGGTTCCACATCGAGGAAGAAAACGCCAGCGGCCCCATGGCCCAGGTCATCGGTCGCATGGAGGCCATCGCCGCCGATACCGGGTGCTCTATCGTGTTCCTGCACCATGCCAGCAAGGGCGCGGCCATGATGGGCGCAGGCGACCAGCAGCAGGCCAGCCGGGGCAGCTCGGTACTGGTCGATAACATCCGCTGGCAGTCCTACCTGTCGAGCATGACCAGCGCCGAGGCCGAGGAATGGGGTGTGGACGACGACCAGCGCCGGTTCTTCGTCCGCTTCGGTGTGAGCAAGGCCAACTATGGCGCACCGTTCGCTGATCGGTGGTTCAGGCGGCATGACGGCGGGGTGCTCAAGCCCGCCGTGCTGGAGAGGCAGCGCAAGAGCAAGGGGGTGCCCCGTGGTGAAGCCTAAGAACAAGCACAGCCTCAGCCACGTCCGGCACGACCCGGCGCACTGTCTGGCCCCCGGCCTGTTCCGTGCCCTCAAGCGGGGCGAGCGCAAGCGCAGCAAGCTGGACGTGACGTATGACTACGGCGACGGCAAGCGGATCGAGTTCAGCGGCCCGGAGCCGCTGGGCGCTGATGATCTGCGCATCCTGCAAGGGCTGGTGGCCATGGCTGGGCCTAATGGCCTAGTGCTTGGCCCGGAACCCAAGACCGAAGGCGGACGGCAGCTCCGGCTGTTCCTGGAACCCAAGTGGGAGGCCGTCACCGCTGATGCCATGGTGGTCAAAGGTAGCTATCGGGCGCTGGCAAAGGAAATCGGGGCAGAGGTCGATAGTGGTGGGGCGCTCAAGCACATACAGGACTGCATCGAGCGCCTTTGGAAGGTATCCATCATCGCCCAGAATGGCCGCAAGCGGCAGGGGTTTCGGCTGCTGTCGGAGTACGCCAGCGACGAGGCGGACGGGCGCCTGTACGTGGCCCTGAACCCCTTGATCGCGCAGGCCGTCATGGGTGGCGGCCAGCATGTGCGCATCAGCATGGACGAGGTGCGGGCGCTGGACAGCGAAACCGCCCGCCTGCTGCACCAGCGGCTGTGTGGCTGGATCGACCCCGGCAAAACCGGCAAGGCTTCCATAGATACCTTGTGCGGCTATGTCTGGCCGTCAGAGGCCAGTGGTTCGACCATGCGCAAGCGCCGCCAGCGGGTGCGCGAGGCGTTGCCGGAGCTGGTCGCGCTGGGCTGGACGGTAACCGAGTTCGCGGCGGGCAAGTACGACATCACCCGGCCCAAGGCGGCAGGCTGACCCCCCCCACTCTATTGTAAACAAGACATTTTTATCTTTTATATTCAATGGCTTATTTTCCTGCTAATCAGCTCGAGGCTTGGATTCTCACCAATAAAAAACGCCCGGCGGCAACCGAGCGTTCTGAACAAATCCAGATGGAGTTCTGAGGTCATTACTGGATCTATCAACAGGAGTCCAAGCGAGCTCGATATCAAATTACGCCCCGCCCTGCCACTCATCGCAGTACTGTTGTAATTCATTAAGCATTCTGCCGACATGGAAGCCATCACAAACGGCATGATGAACCTGAATCGCCAGCGGCATCAGCACCTTGTCGCCTTGCGTATAATATTTGCCCATGGTGAAAACGGGGGCGAAGAAGTTGTCCATATTGGCCACGTTTAAATCAAAACTGGTGAAACTCACCCAGGGATTGGCTGAGACGAAAAACATATTCTCAATAAACCCTTTAGGGAAATAGGCCAGGTTTTCACCGTAACACGCCACATCTTGCGAATATATGTGTAGAAACTGCCGGAAATCGTCGTGGTATTCACTCCAGAGCGATGAAAACGTTTCAGTTTGCTCATGGAAAACGGTGTAACAAGGGTGAACACTATCCCATATCACCAGCTCACCGTCTTTCATTGCCATACGAAATTCCGGATGAGCATTCATCAGGCGGGCAAGAATGTGAATAAAGGCCGGATAAAACTTGTGCTTATTTTTCTTTACGGTCTTTAAAAAGGCCGTAATATCCAGCTGAACGGTCTGGTTATAGGTACATTGAGCAACTGACTGAAATGCCTCAAAATGTTCTTTACGATGCCATTGGGATATATCAACGGTGGTATATCCAGTGATTTTTTTCTCCATTTTAGCTTCCTTAGCTCCTGAAAATCTCGATAACTCAAAAAATACGCCCGGTAGTGATCTTATTTCATTATGGTGAAAGTTGGAACCTCTTACGTGCCCGATCAACTCGAGTGCCACCTGACGTCTAAGAAACCATTATTATCATGACATTAACCTATAAAAATAGGCGTATCACGAGGCAGAATTTCAGATAAAAAAAATCCTTAGCTTTCGCTAAGGATGATTTCTG

**S3 -** Bisabolene production per dry cell weight of all producing *Synechocystis* strains, after 12 days of growth in the MC1000 multicultivators. Values in parenthesis refer to total cumulative yields.

| Strain name | AgB | AgBispA | 2MEP-AgBispA |
| --- | --- | --- | --- |
| Bisabolene yield (mg g^-1^DCW) | 9.3 ± 1.2  (10.2 ± 1.3) | 10.3 ± 1.7  (11.5 ± 2.0) | 15.6 ± 0.9  (17.3 ± 1.0) |

**S4 -** Specific yields of the bisabolene-producing strains after 10 days of cultivation in MC1000 bioreactors and High Cell Density (HCD) system. Values in parenthesis refer to total cumulative yields.


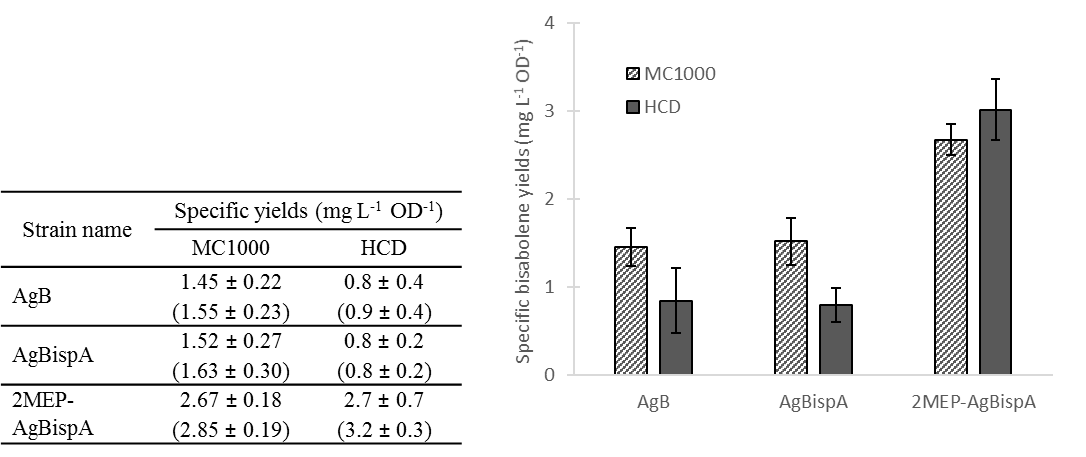

Supplement: Multimedia component 1 [file mmc1.docx]
